# Antimicrobial Peptides for Diabetic Foot Ulcers and Infections: Current Evidence and Translational Perspectives

**DOI:** 10.3390/ijms27188035

**Published:** 2026-09-09

**Authors:** Victoria Alexandrovna Khotina, Arthur Anatolievich Lee, Dmitry Alexandrovich Kashirskikh, Olesya Olegovna Klychkova, Vitalia Sergeevna Novikova, Margarita Pavlovna Markina, Olga Evgenevna Voronko, Vagif Ali oglu Gasanov

**Affiliations:** Koltzov Institute of Developmental Biology of Russian Academy of Sciences, 119334 Moscow, Russia; aal1999arth@gmail.com (A.A.L.); dim.kashirsckih@gmail.com (D.A.K.); olesya_k8@mail.ru (O.O.K.); reddkk@bk.ru (V.S.N.); mmarkina46@gmail.com (M.P.M.); vr_olga@yahoo.com (O.E.V.); gasanovvagif@gmail.com (V.A.o.G.)

**Keywords:** diabetic foot ulcer, diabetic foot infection, antimicrobial peptides, biofilm, wound-targeted delivery, wound healing

## Abstract

Diabetic foot ulcers (DFU) are among the most severe complications of diabetes, resulting from a combination of metabolic dysregulation, vascular insufficiency, neuropathy, chronic inflammation, and impaired tissue repair, whereas diabetic foot infection (DFI) may develop within this compromised wound environment and frequently involves polymicrobial communities and biofilms. This review evaluates the mechanistic and translational basis for the use of antimicrobial peptides (AMP) in DFU and DFI, with emphasis on the diabetic wound microenvironment, polymicrobial ecology, endogenous AMP dysregulation, mechanisms of action, therapeutic development, and barriers to clinical translation. Hyperglycemia, ischemia, oxidative and proteolytic stress, and impaired innate immunity sustain inflammation, delay tissue repair, and promote microbial persistence. These conditions may also complicate antibiotic treatment through impaired tissue exposure and biofilm-associated tolerance. Depending on the peptide and experimental context, AMP may provide direct antimicrobial or antibiofilm activity and may also exert immunomodulatory or pro-reparative effects involving inflammatory signaling, angiogenesis, keratinocyte and fibroblast migration, and re-epithelialization. Approaches under investigation include engineered peptides, combination regimens, and local biomaterial-based platforms, including hydrogels, dressings, scaffolds, and nanoparticle-conjugated systems. Clinical translation remains constrained by proteolytic instability, potential host-tissue toxicity, limited selectivity, limited predictive value of preclinical models, heterogeneous clinical populations, nonstandardized endpoints, and manufacturing and regulatory requirements. Preclinical evidence supports further evaluation of approaches for local delivery of AMP, whereas clinical evidence in DFU and DFI remains limited and heterogeneous, with no AMP-based intervention yet demonstrating sufficiently consistent clinical benefit to support routine use.

## 1. Introduction

Diabetes mellitus (DM) is a chronic, heterogeneous metabolic disorder characterized by persistent hyperglycemia resulting from impaired insulin secretion, reduced tissue sensitivity to insulin, or a combination of these mechanisms [1]. The most common clinical forms are type 1 diabetes mellitus (T1DM) and type 2 diabetes mellitus (T2DM) [1]. The global burden of DM continues to increase and remains a major medical and social challenge worldwide. In 2024, DM affected 588.7 million adults aged 20–79 years globally and was associated with 3.4 million deaths [2]. By 2050, the number of affected individuals is projected to reach 852.5 million, including an increase in Europe from 65.6 to 72.4 million [2].

Chronic hyperglycemia is associated with long-term microvascular and macrovascular complications affecting the eyes, kidneys, peripheral nervous system, and cardiovascular system [3]. These complications manifest as retinopathy, nephropathy, neuropathy, cardiovascular disease, and diabetic foot ulcers (DFU) [3,4]. The spectrum of diabetes-associated complications is broader and also includes nonalcoholic fatty liver disease, cognitive and functional impairment, obstructive sleep apnea, depressive disorders, and certain malignancies [5]. As the prevalence of DM rises, these complications contribute substantially to morbidity, disability, and mortality.

Infectious complications are an important component of diabetes-associated morbidity. Both T1DM and T2DM are associated with an increased risk of lower extremity and foot infections, urinary tract infections, respiratory infections, sepsis, and postoperative infections [4,6]. DFU is particularly clinically consequential because chronic ulceration may progress to diabetic foot infection (DFI), deeper tissue involvement, and amputation. Approximately 18.6 million people with DM are estimated to develop foot ulcers each year worldwide [4,7], and up to approximately 34% of individuals with DM may develop a DFU during their lifetime [4]. A recent systematic review and meta-analysis reported survival rates of 86.9% at 1 year, 66.9% at 3 years, and 50.9% at 5 years in patients with DFU, while infection was the second most common reported cause of death, accounting for a pooled proportion of 24.8% [8]. DFU may also progress to non-traumatic lower extremity amputation, which is associated with mortality rates of up to 70% [4,8].

Susceptibility to ulceration and infection in DM arises from interacting metabolic, vascular, neurological, and immune abnormalities [5,6]. Hyperglycemia promotes formation of advanced glycation end products (AGEs), oxidative stress, chronic inflammation, and dysfunction of innate and adaptive immune responses. Vascular insufficiency further compromises tissue perfusion and may limit delivery of antibacterial agents to sites of infection [3,6]. Hyperglycemia also impairs the function of neutrophils, macrophages, and other immune effector cells [6,9], while diabetic neuropathy reduces sensitivity to repetitive trauma and pressure, promoting persistent tissue injury and ulcer formation [10,11].

Once ulceration occurs, tissue hypoxia, persistent inflammation, impaired perfusion, and defective repair can facilitate microbial persistence and progression to infection. DFI are frequently polymicrobial and may include both aerobic and anaerobic microorganisms. Commonly isolated pathogens include *Staphylococcus aureus*, *Pseudomonas aeruginosa*, *Proteus mirabilis*, *Escherichia coli*, *Klebsiella pneumoniae*, *Enterococcus* spp., and *Corynebacterium* spp. [12,13,14]. Microbial composition varies between patients and clinical settings, while biofilm formation and interactions among members of polymicrobial communities can further contribute to infection persistence.

Antibiotics remain central to the treatment of DFI, but therapeutic response can be complicated by antimicrobial resistance and by wound-related factors that reduce local drug exposure or microbial susceptibility. Resistant pathogens, including methicillin-resistant *S. aureus* (MRSA) and extended-spectrum β-lactam-resistant *E. coli*, are frequently reported in DFI [15]. Ischemia and impaired perfusion may reduce antibiotic delivery to infected tissue [16,17], whereas biofilm growth can increase tolerance to both antimicrobial agents and innate immune defenses [18]. Persistent infection can consequently progress to deeper soft tissues or bone and may ultimately contribute to amputation [16,18].

These limitations have driven the search for alternative or adjunctive anti-infective strategies for DFI. Approaches under investigation include metal- and nanomaterial-based agents, antibacterial polymers, bioactive molecules, and photothermal, photodynamic, and sonodynamic systems [19,20]. Antimicrobial peptides (AMP) represent one group of candidate agents within this broader field. Many naturally occurring AMP are components of innate host defense, whereas other candidates are generated through synthetic and rational-design approaches [21,22]. Individual AMP may exhibit antibacterial, antifungal, or antibiofilm activity and may also influence processes involved in tissue repair by promoting re-epithelialization, angiogenesis, and extracellular matrix (ECM) remodeling [18,23]. Therapeutic development is additionally constrained by proteolytic instability, host–cell toxicity and selectivity, sensitivity to local physicochemical conditions, and manufacturing requirements [21,22].

This review evaluates current evidence for AMP in DFU and DFI, with emphasis on the diabetic wound microenvironment, polymicrobial ecology, endogenous AMP dysregulation, mechanisms of antimicrobial and antibiofilm activity, immunomodulatory and pro-reparative effects, therapeutic development, and barriers to clinical translation. Particular attention is given to the level of evidence supporting individual AMP approaches and to the distinction between mechanistic activity, efficacy in preclinical diabetic-wound models, and clinical outcomes.

## 2. Pathophysiology of the Diabetic Wound Microenvironment

DM is associated with delayed healing of DFU and other chronic wounds, including postoperative wounds, pressure ulcers, and venous leg ulcers (VLU) [24], as well as with increased susceptibility to infection, particularly foot infections and osteomyelitis [6]. In DFU, persistent hyperglycemia, chronic inflammation, vascular insufficiency, peripheral neuropathy, and impaired innate antimicrobial defense disrupt the normal progression of wound healing from inflammation to proliferation and remodeling [6,25]. The resulting wound environment is characterized by tissue hypoxia, oxidative and proteolytic stress, persistent inflammatory activation, and impaired tissue repair, all of which favor microbial persistence and wound chronicity.

### 2.1. Hyperglycemia, Innate Immune Dysfunction, and Inflammatory Persistence

Hyperglycemia is a major determinant of the diabetic wound microenvironment and simultaneously promotes chronic low-grade inflammation, disturbs the transition to the reparative phase, and impairs antimicrobial defense [6,26]. Pattern-recognition receptor signaling pathways involved in this response include TLR2 and TLR4 signaling and activation of the NLRP3 inflammasome, which leads to caspase-1 activation and subsequent maturation and secretion of IL-1β and IL-18, thereby sustaining proinflammatory signaling [26].

Hyperglycemia also promotes accumulation of AGEs, which contribute to tissue injury and impaired repair [27]. In db/db mice, dietary AGE restriction reduced AGE deposition in the skin and was associated with improved epithelialization, angiogenesis, collagen organization, reduced inflammation, and faster wound closure [27]. In this model, these findings link glycation-related metabolic stress to impaired wound repair.

The complement system is also altered under hyperglycemic conditions. In serum-based experimental assays, elevated glucose reduced C3 activation, C3b and iC3b binding to the surface of *S. aureus*, C5a generation, and serum-mediated neutrophil phagocytosis [28]. In a serum-based polymicrobial model under elevated-glucose conditions, C3 depletion was associated with reduced complement-mediated killing of *E. coli* [28]. Thus, hyperglycemia can impair both pathogen opsonization and downstream cellular clearance.

Neutrophil dysfunction further compromises antimicrobial defense in DM [29]. Neutrophil functions required for pathogen elimination, including migration, chemotaxis, phagocytosis, and intracellular reactive oxygen species (ROS) generation, are impaired, resulting in reduced bactericidal activity [29]. At the same time, extracellular ROS production, formation of neutrophil extracellular traps (NETs), and release of proinflammatory mediators may remain increased, contributing to tissue injury and persistent inflammation [29]. This combination reduces microbial clearance while maintaining inflammatory damage.

In patients with DFU, systemic inflammatory markers, including C-reactive protein, fibrinogen, and IL-6, are elevated, and inflammatory burden appears to correlate more strongly with ulcer severity than with infection status alone [30,31]. Persistent inflammatory signaling also interferes with the macrophage transition from the proinflammatory M1 phenotype to the reparative M2 phenotype and contributes to wound chronicity in DM [32,33,34]. In *P. aeruginosa*-infected diabetic wounds in db/db mice, infection was associated with increased *Tnf-α*, *Il-1β*, and *Il-6* gene expression and prolonged persistence of M1 macrophages within the wound [35]. Although M2-associated markers, including IL-10, arginase-1, and YM1, were also increased, the response remained predominantly inflammatory and was accompanied by reduced re-epithelialization, impaired granulation tissue formation, and defective angiogenesis [35]. Overall, hyperglycemia creates a wound environment in which impaired microbial clearance coexists with persistent inflammatory activation.

### 2.2. Infection and Biofilm as Amplifiers of Wound Chronicity

Ulceration disrupts the cutaneous barrier and exposes underlying tissue to microbial contamination, while hyperglycemia, ischemia, neuropathy, and impaired innate immunity facilitate progression toward persistent infection. Experimental models of infected diabetic wounds indicate that pathogens can further impair healing through distinct but convergent immune mechanisms.

In diabetic TALLYHO mice with *S. aureus* biofilm-containing wounds, reduced TLR2 and TLR4 expression, lower local IL-1β and TNF-α levels, impaired neutrophil entry into regions containing bacterial aggregates, and reduced myeloperoxidase activity were associated with higher bacterial burden and delayed epithelialization [36]. These findings indicate that, in this model, biofilm persistence in diabetic wounds was accompanied by impaired pathogen recognition, restricted neutrophil access, and defective oxidative killing. In streptozotocin (STZ)-induced diabetic mice with *S. aureus* skin infection, excessive macrophage and neutrophil infiltration coexisted with defective abscess organization, altered cytokine and chemokine expression, and increased bacterial burden [37]. Elevated cutaneous LTB4/BLT1 signaling contributed to this disorganized inflammatory response, whereas BLT1 genetic deletion or pharmacological inhibition improved local immune organization and antimicrobial defense [37].

Together, these experimental findings indicate that infection and biofilm formation can reinforce diabetic wound chronicity by combining ineffective microbial clearance with persistent inflammatory activation, thereby delaying progression toward tissue repair [10].

### 2.3. Neurovascular Insufficiency and Failure of Tissue Repair

Hyperglycemia-associated vascular dysfunction contributes to ischemia and tissue hypoxia through oxidative stress, AGE–RAGE signaling, endothelial injury, and reduced nitric oxide bioavailability [34]. At the macrovascular level, these changes contribute to peripheral arterial disease, whereas at the microvascular level, vascular basement membrane thickening, impaired vascular dilation, reduced perfusion, and defective angiogenic responses impair oxygen diffusion and limit compensatory revascularization within the wound bed [34]. Additionally, hyperglycemia, dyslipidemia, insulin resistance, and oxidative stress impair endothelial cell proliferation and migration and increase apoptosis [34,38]. In turn, endothelial cell dysfunction and impaired regenerative responses further limit compensatory angiogenesis in diabetic wounds [16]. The resulting hypoxic and nutrient-deprived environment delays re-epithelialization, granulation tissue formation, and bacterial clearance and promotes persistent tissue injury.

Neuropathy is observed in 30–50% of patients with diabetes and further promotes ulcer persistence by reducing protection against repetitive mechanical injury [25]. Sensory neuropathy results in loss of pain, temperature, and tactile sensation, allowing repetitive pressure, microtrauma, and minor injury to persist unnoticed [31]. Motor neuropathy causes muscle atrophy and imbalance, altering plantar loading and generating focal areas of increased pressure that predispose to recurrent tissue injury [16]. Autonomic neuropathy contributes to anhidrosis, skin dryness and fissuring, and impaired barrier function [39]. Arteriovenous shunting and impaired neurogenic control of vascular tone further aggravate microcirculatory and trophic abnormalities [16,39]. These abnormalities facilitate continued tissue injury and microbial entry into an already poorly perfused wound environment.

Neurovascular dysfunction is accompanied by impaired cellular repair. High glucose impairs keratinocyte function and delays re-epithelialization, while hyperglycemia and AGE accumulation suppress fibroblast proliferation and migration, increase apoptosis, and limit formation of mature granulation tissue [40]. Because fibroblasts are required for ECM synthesis, dermal remodeling, and differentiation into myofibroblasts, their dysfunction directly compromises wound closure [40]. Thus, coexisting ischemia and neuropathy combine tissue hypoxia, impaired microcirculation, defective angiogenesis, barrier disruption, and reduced reparative capacity, creating conditions that favor chronic inflammation, infection, and nonhealing DFU [16,34,39].

### 2.4. Oxidative, Proteolytic, and Exudative Barriers to Healing

The diabetic wound microenvironment is characterized by oxidative and proteolytic factors that sustain tissue injury and delay transition to the proliferative phase. Hyperglycemia, hypoxia, chronic inflammation, and vascular dysfunction increase ROS generation and impair antioxidant defenses [16,41,42]. AGE-RAGE signaling further amplifies inflammation and ROS generation, promoting lipid peroxidation and DNA damage, and contributing to cellular injury and defective tissue repair [16]. In patients with nonhealing DFU, increased lipid peroxidation and reduced total antioxidant status support the clinical relevance of persistent oxidative injury [42].

Chronic inflammation and immune dysregulation also contribute to increased proteolytic activity within diabetic wounds. Neutrophils and other inflammatory cells release proteases, cathepsins, and matrix-degrading enzymes, including matrix metalloproteinases (MMPs). Although MMPs participate in physiological ECM turnover and wound remodeling, persistent elevation of MMPs, particularly MMP-9, and disruption of the MMP/TIMP balance are associated with impaired DFU healing [43].

Wound exudate in DFU contains cytokines, proteases, ROS, and products of oxidative injury that can sustain tissue damage and reduce growth factor activity [34,41]. Within this environment, endothelial cells, keratinocytes, and fibroblasts show reduced survival and function, while neovascularization and recruitment of reparative cells are impaired [34]. The high proteolytic activity and complex composition of chronic wound fluid also create conditions in which locally active peptide mediators may undergo degradation, binding, or functional inactivation. Thus, oxidative stress, proteolysis, and exudate composition reinforce both defective tissue repair and instability of local host-defense mechanisms.

### 2.5. Dysregulation of Endogenous Antimicrobial Peptides in Diabetes

Dysregulation of endogenous AMP may contribute to impaired antimicrobial defense and delayed wound repair in DM. Major cutaneous AMP include α-defensins, β-defensins (hBDs), the cathelicidin LL-37, and dermcidin, which are produced by resident skin cells and infiltrating immune cells [44,45]. In addition to direct antimicrobial activity, these peptides can influence inflammatory signaling, angiogenesis, and tissue repair by supporting the proliferation, migration, and differentiation of fibroblasts and keratinocytes [46,47,48,49].

Human DFU tissue shows selective rather than uniform changes in AMP expression. In Wagner grade 3 DFU biopsies, genes encoding hBD-2, hBD-3, and hBD-4 were upregulated, whereas *CAMP* expression was markedly reduced or absent compared with healthy skin [50]. Primary keratinocytes derived from DFU also showed lower LL-37 expression after infection with *S. aureus* compared with keratinocytes from healthy donors [50]. Reduced expression of genes *CAMP* and *DEFB4* has additionally been reported in peripheral blood cells and epidermal keratinocytes derived from DFU [51,52]. These findings indicate that endogenous AMP expression is not uniformly reduced in DFU, but shows peptide-specific alterations. The coexistence of increased β-defensin expression with reduced *CAMP*/LL-37 expression further indicates nonuniform AMP regulation in DFU [50].

Local expression of α-defensins, known as human neutrophil peptides (HNPs), is also heterogeneous in DFU tissue. HNP-1, HNP-3, and HNP-4 expression levels were higher in the ulcer center than at the wound edge and were accompanied by increased IL-8 levels in wound exudate [53]. In contrast, immunohistochemical analysis showed no overall difference in HNP-1 staining between DFU tissue and healthy skin, although a subset of patients had marked neutrophilic dermal infiltration with intense HNP-1 staining and elevated *DEFA1* expression [50]. These data support a focal, inflammation-associated pattern of α-defensin activation rather than uniform upregulation throughout the wound.

Systemic studies further support nonuniform AMP regulation in diabetes. Serum α-defensin levels are increased in patients with T1DM or T2DM compared with healthy controls and have been associated with metabolic and inflammatory indices [54,55]. In contrast, plasma LL-37 levels in T2DM were more closely associated with markers related to systemic inflammatory status than with glycemic control [56]. More directly relevant to diabetic foot infection, plasma dermcidin levels were lower in patients with DFI than in diabetic patients without previous DFI and in healthy controls [57].

Overall, available human data indicate that endogenous AMP regulation in diabetes is tissue- and context-dependent rather than characterized by uniform deficiency. In DFU and DFI, reduced local LL-37 expression, lower plasma dermcidin levels, focal neutrophil-associated α-defensin activation, and variable β-defensin responses indicate nonuniform remodeling of endogenous antimicrobial defense. Endogenous AMP dysregulation in the diabetic wound therefore appears to reflect broader alterations in local innate defense rather than a simple quantitative deficiency.

## 3. Polymicrobial Ecology of Diabetic Foot Infection

Disruption of the skin barrier, hyperglycemia, impaired innate immune defense, neuropathy, and ischemia promote microbial colonization of DFU and progression to infection. DFU may therefore contain complex microbial communities in which microbial composition, host–microbe interactions, interspecies relationships, and biofilm formation contribute to infection persistence and impaired wound healing [10]. DFI occurs in approximately 60% of DFU cases and remains one of the most frequent complications of DM, with severe infection associated with an increased risk of amputation [8].

### 3.1. Microbiological Composition and Geographic Heterogeneity of Diabetic Foot Infection

The microbiological profile of DFI varies with ulcer depth, infection severity, duration of wound persistence, prior antibacterial therapy, and geographic region. The most commonly detected pathogens include aerobic Gram-positive bacteria such as *Staphylococcus* spp. (*S. aureus*, *S. epidermidis*), *Corynebacterium* spp., *Enterococcus* spp. (*E. faecalis*), *Streptococcus* spp. (*S. agalactiae*), together with Gram-negative bacteria including *Pseudomonas* spp. (*P. aeruginosa*), *Escherichia* spp. (*E. coli*), *Enterobacter* spp., *Proteus* spp. (*P. mirabilis*), *Serratia* spp., *Acinetobacter* spp. (*A. baumannii*), *Klebsiella* spp. (*K. pneumoniae*) and *Stenotrophomonas* spp. (*S. maltophilia*) [10,12,13,14,15,24,58]. In deeper and more chronic infections, particularly in the presence of necrosis and longstanding ulcers, anaerobes are also frequently detected, including Gram-positive *Clostridium* spp. (*C. perfringens*, *C. sporogenes*, *C. tetanomorphum*, *C. subterminale*), *Peptostreptococcus* spp. (*P. asaccharolyticus*), and, less commonly, *Finegoldia* spp. (*F. magna*) and *Anaerococcus* spp. (*A. lactolyticus*, *A. obesiensis*, *A. vaginalis*), as well as Gram-negative *Bacteroides* spp. (*B. fragilis*, *B. xylanisolvens*), *Prevotella* spp. (*P. intermedia*, *P. melaninogenica*, *P. scopos*, *P. bivia*), *Porphyromonas* spp. (*P. asaccharolytica*), *Fusobacterium* spp. (*F. nucleatum*), and *Veillonella* spp. (*V. parvula*) [10,24,59]. Notably, anaerobe-associated DFI has been linked to a higher risk of amputation [59,60].

Regional studies show substantial variation in microbial composition, polymicrobial burden, and antimicrobial resistance patterns. Several South Asian cohorts report a predominance of Gram-negative bacteria, whereas some European cohorts remain dominated by Gram-positive organisms, particularly *S. aureus* [61,62,63,64,65,66]. Clinical data from the Middle East and North Africa show mixed patterns, with either staphylococcal predominance or aerobic Gram-negative bacilli, often within polymicrobial infections [67,68,69]. Sequencing-based studies detect greater microbial diversity and a larger anaerobic component than conventional culture, indicating that routine culture may incompletely characterize the microbial community and virulence-related features [60]. Cross-regional comparisons should be interpreted cautiously because the available studies differ in sample size, patient selection, ulcer and infection severity, previous antimicrobial exposure, sampling strategy, microbiological methods, and definitions of polymicrobial infection [60,61,62,63,64,65,66,67,68,69,70,71]. The proportions summarized below are therefore descriptive and should not be interpreted as directly comparable between regions. Representative regional patterns are summarized in Table 1.

Fungal involvement has also been reported in DFU, with some studies detecting fungi in up to 40% of diabetic wounds [18,72]. The fungal taxa most often associated with DFU include *Candida* spp. (*C. albicans*, *C. parapsilosis*, *C. tropicalis*, *C. guilliermondii*), *Trichophyton* spp. (*T. rubrum*, *T. mentagrophytes*), *Aspergillus* spp. (*A. flavus*, *A. niger*), *Trichosporon* spp., *Fusarium* spp. (*F. acutatum*) and, less frequently, *Cladosporium* spp. and the recently described *Coniochaeta diabeticopedicola* [10,72,73]. Fungal involvement and mixed bacterial–fungal communities have been reported in chronic or poorly healing DFU, although their clinical significance varies across studies. Because fungi and anaerobic bacteria are often underdetected by standard diagnostic approaches, infectious community structure may be incompletely characterized [10,18,59,72,73]. Fungal elements may also provide a scaffold for bacterial adhesion and support formation of mixed-kingdom biofilms, increasing tolerance to antimicrobial interventions [10].

### 3.2. Polymicrobial Interactions and Biofilm Organization in DFI

Chronic and clinically significant DFI frequently involves polymicrobial communities in which microbial interactions may contribute to infection persistence rather than reflecting a simple accumulation of independent species. Aerobic and anaerobic bacteria, and, in some cases, fungi, may co-aggregate within a shared matrix and form functionally equivalent pathogroups, in which pathogenic and opportunistic commensal organisms contribute collectively to infection persistence [17]. Cross-feeding, metabolic niche partitioning, shared virulence factors, and amplification of stress-tolerance mechanisms can support survival of the community.

Biofilm formation is a major organizational feature of these communities [17,18]. In DFU, biofilms consist of structured microbial aggregates embedded in an extracellular polymeric substance matrix composed of polysaccharides, proteins, and extracellular DNA [17,24]. Biofilm formation is accompanied by changes in microbial growth, metabolism, stress responses, and gene expression and may also be associated with enhanced virulence, while close spatial organization may facilitate horizontal gene transfer [17]. Biofilm formation is therefore a central mechanism linking colonization to persistent, difficult-to-treat infection in DFU [17,18].

Biofilm-associated tolerance is not limited to matrix-mediated physical protection. Compared with planktonic organisms, bacteria within biofilms are less susceptible to host immune clearance and antibiotic treatment because the matrix restricts drug diffusion, microbial metabolism becomes heterogeneous, persister cells emerge, and some species become spatially shielded by others [24]. In polymicrobial biofilms, these effects are reinforced by interspecies cooperation, including modification of local pH, nutrient or oxygen availability, matrix stabilization, quorum sensing, and coordinated stress responses [17,24].

### 3.3. Biofilm-Associated Tolerance, Antimicrobial Resistance, and Adverse Clinical Outcomes

Clinical and microbiological studies associate biofilm formation, polymicrobial infection, multidrug resistance (MDR), and adverse outcomes in DFI. In chronic diabetic wounds, biofilm represents a functional state of the infectious community that modifies therapeutic response. Reduced susceptibility to antibiotics and innate immune effectors may be reinforced by cooperative interactions that preserve community-level tolerance even when resistance determinants are unevenly distributed among constituent species [17,24,71]. For example, in mixed-species macrocolony biofilms generated from clinical isolates obtained from polymicrobial DFU, *E. faecalis* showed approximately 1-log higher cell counts in combination with *E. coli* and 2–4-log higher counts with *P. aeruginosa* than in corresponding single-species biofilms [71]. The resistance and biofilm data summarized in Table 2 should likewise be interpreted descriptively. Differences in susceptibility-testing methods, definitions of MDR and biofilm positivity, sampled populations, antimicrobial exposure, and microbiological methods preclude direct quantitative comparison between regional cohorts.

Despite geographic variation in taxonomic composition, regional studies repeatedly report biofilm formation, polymicrobial infection, and antimicrobial resistance, although their prevalence cannot be directly compared across cohorts. In staphylococcal-predominant cohorts, infection persistence has been associated with biofilm-forming capacity and methicillin resistance, whereas Gram-negative-enriched communities frequently include ESBL-, AmpC β-lactamase-, or carbapenemase-producing organisms (Table 2). Sequencing-based data further indicate that adverse outcomes may be associated with community-level features, including genes related to biofilm formation, antibiotic resistance, and bacterial virulence [60]. Thus, therapeutic response may depend on both taxonomic composition and the functional organization of the microbial community.

The effectiveness of antibiotic therapy in DFI is influenced by both microbial and wound-related factors. Ischemia, reduced perfusion, tissue hypoxia, and impaired drug delivery may reduce antibiotic exposure at the site of chronic infection [17]. Polymicrobial organization and underdetection of anaerobes and fungi by standard culture complicate identification of clinically relevant pathogens and may lead to inadequate empirical therapy [18,60]. Repeated or inappropriate antibiotic exposure further selects resistant organisms, including MRSA, ESBL-producing *E. coli* and other Enterobacteriaceae, metallo-β-lactamase-producing *K. pneumoniae*, and fluoroquinolone-resistant isolates [12,15,67]. Antibiotic activity within chronic DFU is influenced not only by conventional antimicrobial susceptibility but also by biofilm-associated factors, including restricted drug penetration, high microbial density, metabolic heterogeneity, persister cells, and interspecies interactions [10,24].

Collectively, available studies characterize DFI as microbiologically heterogeneous and frequently associated with polymicrobial organization, biofilm formation, and antimicrobial resistance. These features define important conditions for evaluating anti-infective therapies, including activity against biofilm-associated bacteria, resistant organisms, anaerobes, and mixed bacterial–fungal communities [16,18].

## 4. Mechanistic Basis of AMP Activity in Diabetic Wounds and Infection

AMP comprise a structurally diverse group of peptides involved in innate host defense and antimicrobial activity [22]. They are commonly 12–50 amino acid residues in length and are often cationic and amphipathic, with activity influenced by net charge, hydrophobicity, molecular length, and secondary structure [22,74]. Despite their structural diversity, these physicochemical properties facilitate interactions with microbial membranes and can also contribute to intracellular, immunomodulatory, and reparative effects.

**Figure 1:**
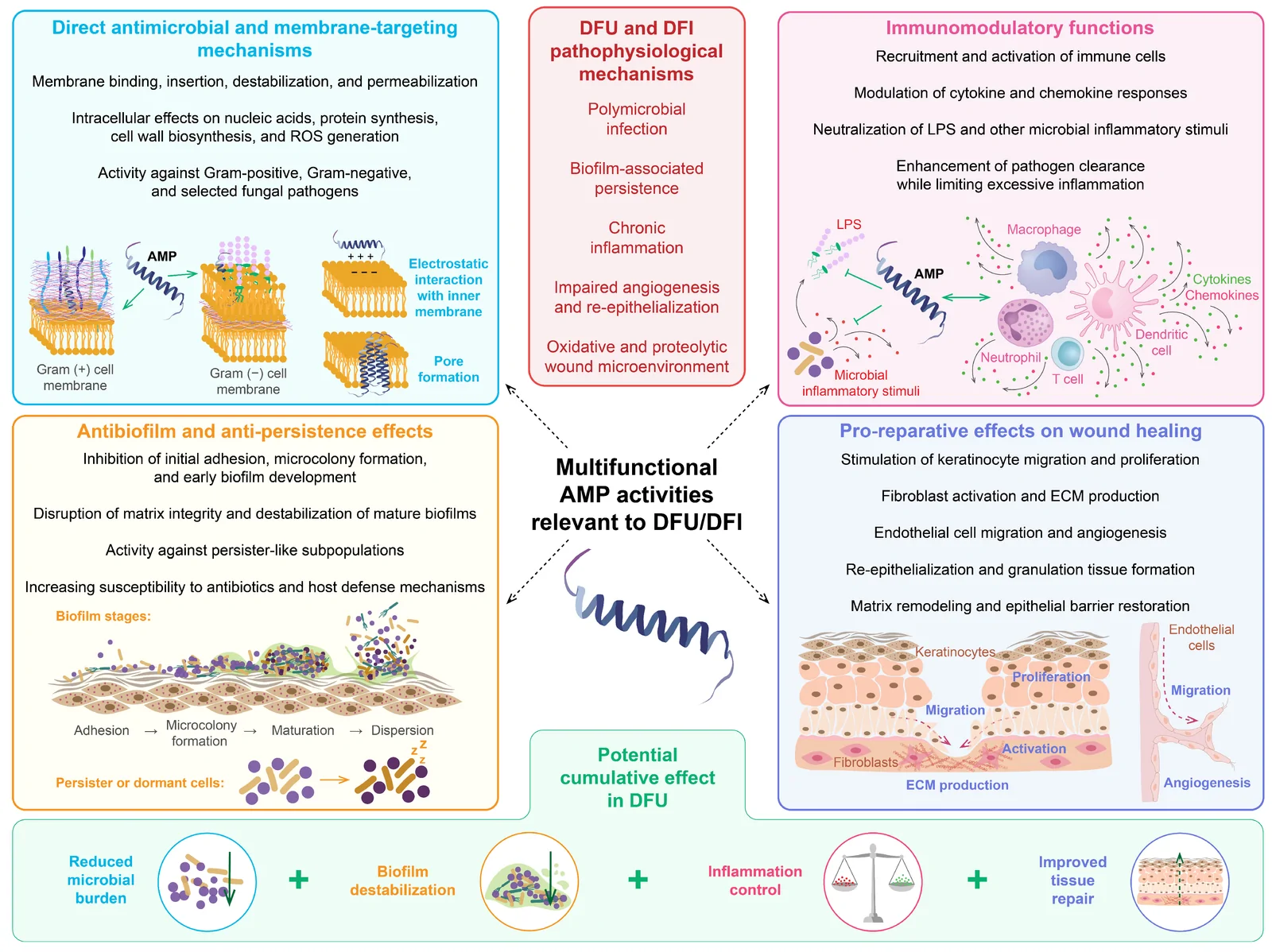
Multifunctional activities of antimicrobial peptides relevant to DFU and DFI. The central panel summarizes major pathophysiological features of the diabetic wound environment, whereas the surrounding panels illustrate distinct antimicrobial, antibiofilm, immunomodulatory, and pro-reparative activities of selected AMP. The lower panel depicts their potential cumulative effects on microbial control, inflammation, and tissue repair. These activities are peptide- and context-dependent.

Based on secondary structure, AMP can be classified into four major groups: (1) α-helical; (2) β-sheet; (3) αβ; (4) non-αβ peptides [22,74]. Non-αβ AMP lack a predominant α-helical or β-sheet structure and include extended or loop peptides, such as tryptophan-rich, proline-rich, and glycine-rich peptides [74]. Human α- and β-defensins are predominantly disulfide bond-stabilized β-sheet peptides, whereas LL-37 is a representative α-helical cathelicidin [22,74]. This structural diversity is associated with different mechanisms of action, including direct microbial killing, interference with biofilm-associated phenotypes, modulation of host inflammatory responses, and effects on tissue repair (Figure 1).

### 4.1. Direct Antimicrobial Mechanisms and Broad-Spectrum Activity

For many AMP, initial binding involves electrostatic interactions with negatively charged components of the microbial envelope, including lipopolysaccharides (LPS) in Gram-negative bacteria and lipoteichoic acid and peptidoglycans in Gram-positive bacteria [22,75]. Cationic charge and amphipathicity facilitate membrane binding and insertion, which can result in pore formation, bilayer destabilization, loss of membrane integrity, and leakage of intracellular contents [22,75]. Compared with bacterial membranes, mammalian cell membranes generally have a lower net negative charge and a higher cholesterol content, which can reduce susceptibility to membrane-disruptive AMP [75].

AMP may also act through non-membrane or intracellular targets. Following surface binding and translocation, some peptides interact with nucleic acids, inhibit protein or cell wall component synthesis, interfere with enzyme activity, interact with heat shock proteins, or alter cellular redox homeostasis [75]. For example, hBD-3 interferes with cell wall biosynthesis in staphylococci [76]. At low non-permeabilizing concentrations, HNP-1, HNP-4, hBD-2, and hBD-3 inhibited plasma membrane H+-ATPase activity in *P. aeruginosa* and *C. albicans* in vitro, with intracellular acidification implicated in microbial killing [77]. Against MDR Gram-negative clinical isolates, hBD-3 showed measurable bactericidal activity, particularly against *A. baumannii* and *E. cloacae*, and the observed effects appeared to depend predominantly on membrane permeabilization rather than peptidoglycan targeting [78]. LL-37 can interact with intracellular bacterial biomacromolecules, including DNA and ribosomes, after cytoplasmic entry [79]. Experimental cathelicidin peptides can also induce bacterial ROS accumulation, which contributes to growth inhibition and cellular damage [80].

Some AMP also exhibit antifungal activity. LL-37 induces oxidative stress and disrupts cell-cycle progression in *Candida auris* in vitro [81]. Histatins, a family of histidine-rich salivary peptides, also display antimicrobial and antifungal activity [82]. The best-studied members of this group include histatin-1 (Hst1), histatin-2 (Hst2), histatin-3 (Hst3), and histatin-5 (Hst5). Among histatins, Hst5 shows the highest antifungal activity and has demonstrated in vitro activity against *C. albicans*, *C. krusei*, *C. glabrata*, *Cryptococcus neoformans*, and *Aspergillus fumigatus* [82]. In *C. albicans*, Hst5-induced killing has been associated with mitochondrial dysfunction and reduced ATP synthesis, consistent with bioenergetic collapse [83]. In turn, the Hst5-derived fragment P-113 can penetrate *S. mutans* and accumulate intracellularly, where its antimicrobial mechanism may involve DNA binding [84]. These findings extend the mechanistic spectrum of AMP activity beyond bacteria, although activity against individual fungal species does not itself establish efficacy against mixed bacterial–fungal communities in DFI.

### 4.2. Biofilm Disruption and Activity Against Persistent Microbial Phenotypes

AMP can interfere with several stages of biofilm development, including microbial adhesion, microcolony formation, maturation, quorum sensing, and other biofilm-regulatory pathways [85,86]. Their effects may involve membrane disruption, inhibition of initial adhesion and microbial aggregate formation, and modulation of microbial stress responses [87]. These mechanisms differ from planktonic antimicrobial activity because biofilm-associated microorganisms are exposed to restricted diffusion, heterogeneous nutrient and oxygen availability, altered metabolic states, and persister-cell formation.

Antibiofilm efficacy also depends on the physiological state and maturity of the microbial community. Nisin Z, a natural antimicrobial peptide (lantibiotic) produced by *Lactococcus lactis*, inhibited *P. aeruginosa* persisters isolated from DFI and partially reversed persistence-associated changes in metabolic, stress-response, and biofilm-related pathways [88]. However, it did not fully eradicate persisters within established mature biofilms, indicating that activity against planktonic or persister populations does not necessarily predict activity against mature biofilm communities. LL-37 has likewise been shown to inhibit *S. aureus* adhesion and to exert antistaphylococcal activity against mature biofilms in static in vitro biofilm models [87]. hBD-3 also inhibited adhesion, biofilm formation, and early biofilm maturation of methicillin-resistant *Staphylococcus* strains on titanium surfaces in vitro [89]. Antibiofilm activity has also been demonstrated against fungal targets. Hst5 inhibited biofilm metabolic activity and yeast-to-hyphae transition and reduced biofilm thickness in a fluconazole-resistant *C. albicans* clinical isolate in vitro [90].

Antibiofilm activity is therefore mechanistically distinct from conventional planktonic killing and varies with peptide properties, microbial species, community composition, and biofilm maturity. This distinction is particularly relevant to polymicrobial and mixed bacterial–fungal communities, in which interspecies interactions can further modify antimicrobial susceptibility [10,18,73].

### 4.3. Immunomodulatory Functions Relevant to the Diabetic Wound Microenvironment

In addition to direct antimicrobial effects, AMP can regulate innate and adaptive immune responses. This activity is relevant to DFU, where persistent inflammation coexists with impaired microbial clearance and defective transition to tissue repair.

Several peptides, including hBD-2, hBD-3, LL-37, and pro-dermcidin, the 110-amino-acid precursor of dermcidin, influence leukocyte recruitment, activation, and cytokine production [45,91]. Human α-defensins selectively attract CD4+ CD45RA+ naive T cells, β-defensins can recruit resting memory T cells and immature dendritic cells, and LL-37 can recruit neutrophils, monocytes, mast cells, and T cells [91]. AMP can also modify cellular responses to TLR ligands, promote leukocyte differentiation, and interact with bacterial components such as LPS and lipoteichoic acid, thereby affecting both pathogen recognition and inflammatory signaling [91].

LL-37 illustrates the concentration- and context-dependent nature of AMP immunomodulation. Its expression is regulated in part through TLR-dependent activation of the vitamin D pathway, which induces *CAMP* expression in monocytes, macrophages, and epithelial cells [92]. At low concentrations (<5 μg/mL), LL-37 did not substantially activate monocytes in isolation but induced MAPK/ERK-dependent monocyte activation in an epithelial–immune coculture model containing a polarized Caco-2 monolayer [93]. Under these coculture conditions, LL-37 increased the production of several inflammatory and regulatory mediators, including G-CSF, GM-CSF, M-CSF, IL-1β, TNF-α, and IL-10 [93].

A similarly context-dependent response has been observed in primary human keratinocytes. LL-37 increased secretion of CXCL8 and IL-1β under unstimulated, Th1-like, and Th17-like conditions, while additional effects on CXCL2, GM-CSF, VEGF, and IL-6 were observed depending on the surrounding cytokine environment [94]. The broader secretory response also differed between Th1- and Th17-like conditions, indicating that LL-37 can modify rather than uniformly suppress or amplify inflammatory signaling [94].

Complex formation between LL-37 and nucleic acids provides an additional mechanism of immunomodulation. LL-37 can associate with extracellular DNA and RNA, including nucleic acids released within NETs, and facilitate their interaction with nucleic-acid-sensing receptors such as TLR3, TLR7, TLR8, and TLR9 [79,91]. Depending on the nature and localization of the nucleic acid, these complexes may contribute to antimicrobial host defense or promote sustained inflammatory signaling [79].

Dermcidin-related host defense may likewise involve host–cell mechanisms in addition to direct antimicrobial activity. Although the C-terminal antimicrobial domains of dermcidin can exhibit direct bactericidal activity, full-length pro-dermcidin does not directly kill *E. coli* in macrophage-based studies but increases LC3-II formation and LC3 puncta, consistent with activation of LC3-associated phagocytic or autophagic pathways [45]. These effects may enhance bacterial clearance through modulation of macrophage function rather than direct membrane disruption.

Overall, AMP immunomodulatory activity is peptide-, concentration-, cell-, and microenvironment-dependent. Antimicrobial potency therefore does not necessarily predict the direction or magnitude of host inflammatory responses.

### 4.4. Pro-Reparative Mechanisms Relevant to Chronic Wound Healing

Several AMP influence processes required for wound repair, including angiogenesis, keratinocyte and fibroblast migration, re-epithelialization, and barrier restoration. These effects are mechanistically distinct from infection control and therefore require separate evaluation.

Recombinant P-LL37 induced endothelial cell proliferation, migration, and formation of tubule-like structures in vitro, whereas topical synthetic and recombinant LL-37 increased vascularization and re-epithelialization in wounds in non-diabetic dexamethasone-treated mice in vivo [95]. Its angiogenic activity appears to involve both direct endothelial effects and context-dependent regulation of angiogenic mediators. In human umbilical vein endothelial cells (HUVECs), LL-37 directly stimulated proliferation and vascular structure formation without increasing VEGF secretion, indicating that this endothelial response can occur independently of VEGF [46]. In contrast, increased hCAP-18/LL-37 expression in HaCaT keratinocytes was associated with increased VEGF mRNA and protein and accumulation of HIF-1α protein [96]. Importantly, expression of hCAP-18/LL-37 in full-thickness wounds in diabetic db/db mice increased VEGFA expression and accelerated re-epithelialization [97]. In a hind-limb ischemia model, hCAP-18/LL-37 expression also increased VEGFA and SDF-1α signaling and improved tissue perfusion [97].

More recent studies have extended these observations. Exogenous LL-37 increased HUVEC proliferation, migration, and tube formation together with VEGFA expression and activation of PI3K/AKT/mTOR signaling; in a non-diabetic mouse hind-limb ischemia model, LL-37 also improved perfusion and increased CD31 and CD34 expression [98]. In a full-thickness wound model in diabetic mice, LL-37 accelerated wound healing through TFEB-dependent autophagy, while under high-glucose conditions in vitro it stimulated migration of HaCaT keratinocytes [99].

Human β-defensins also modulate skin-cell migration, inflammatory signaling, and angiogenic responses. hBD-2 stimulated fibroblast migration, whereas hBD-3 induced cytokine and chemokine expression in keratinocytes in vitro [100]. In primary human keratinocytes, hBD-2, hBD-3, and hBD-4 increased both gene expression and protein production of IL-6, IL-10, IP-10 (CXCL10), MCP-1 (CCL2), MIP-3α (CCL20), and RANTES (CCL5) and promoted keratinocyte migration and proliferation [48]. hBD-1, hBD-2, hBD-3, and hBD-4 dose-dependently increased angiogenin secretion by human dermal fibroblasts in two-dimensional culture and a three-dimensional type I collagen-gel model through EGFR-, Src-, JNK-, p38-, and NF-κB-dependent pathways [101]. Platelet-released growth factors increased hBD-3 expression in primary keratinocytes in vitro, partly through EGFR signaling, whereas Vivostat platelet-rich fibrin enhanced hBD-3 expression in artificially generated skin wounds in healthy human volunteers in vivo [102].

hBD-2 may additionally influence epithelial barrier integrity. In a HaCaT monolayer model of *S. aureus* V8 (SspA)-mediated proteolytic barrier damage, exogenous hBD-2 reduced barrier injury without directly inhibiting V8 protease or altering keratinocyte migration and proliferation in undamaged monolayers [103]. Under proteolytic exposure, hBD-2 altered proteomic and secretomic profiles, including ECM-associated proteins, consistent with a damage-context-dependent barrier-protective response [103].

Histatins also exhibit pro-reparative activity in skin-related experimental models. Hst2 promoted primary skin keratinocyte migration and re-epithelialization in a human full-skin-equivalent wound model in vitro [104]. Promigratory effects of Hst2 were also observed in dermal fibroblasts in vitro [104]. Hst1 promoted endothelial adhesion, spreading, migration, and tube formation in vitro, whereas endothelial migration required activation of the RIN2/Rab5/Rac1 signaling axis associated with vascular morphogenesis and angiogenesis [82]. Under oxidative stress in HUVECs, Hst1 reduced cellular senescence through NOX/ERK/Nrf2-associated antioxidant signaling and inhibited IP3R1/GRP75/VDAC1-mediated mitochondria-associated ER membrane formation, thereby limiting mitochondrial Ca^2+^ overload and dysfunction [105]. In an STZ-induced diabetic mouse wound model, Hst1 promoted wound healing and neovascularization while reducing the expression of senescence-associated markers [105]. In high-glucose-injured human keratinocytes in vitro, Hst1 increased proliferation and reduced apoptosis; these effects were associated with modulation of Ras–Raf–MEK–ERK/MAPK signaling, including reduced MEK phosphorylation [106]. Hst5 also promoted epithelial cell migration in vitro and accelerated wound closure in a murine corneal injury model, indicating that its biological activity extends beyond its established antifungal effects [107].

Collectively, these studies show that selected AMP can influence angiogenesis, cell migration, barrier integrity, and re-epithelialization in cell-based and animal wound models. These pro-reparative effects should be distinguished from direct antimicrobial activity, and neither mechanistic activity alone establishes clinical wound-healing efficacy.

### 4.5. Multifunctional Activities of AMP in the Diabetic Wound Microenvironment

Multifunctionality is a characteristic of several AMP, whose biological effects extend beyond direct microbial killing and may involve host–cell responses relevant to wound repair. In the diabetic wound microenvironment, a single peptide may combine antimicrobial or antibiofilm activity with modulation of inflammatory signaling, cell migration, angiogenesis, or epithelial barrier restoration. The relative contribution of these functions varies with peptide concentration, microbial target, cell type, and local wound conditions.

LL-37 illustrates this functional overlap. The same peptide exhibits direct antimicrobial activity while also modulating inflammatory responses and promoting endothelial and keratinocyte functions associated with angiogenesis and wound repair in experimental systems, including diabetic wound models [46,95,99].

Human β-defensins similarly combine antimicrobial activity with effects on keratinocyte migration, cytokine and chemokine production, barrier integrity, and angiogenic signaling [48,101,103]. This functional overlap has also been demonstrated in *S. aureus*-infected wounds in STZ-induced diabetic Yorkshire pigs, in which hBD-3 expression reduced bacterial burden on day 4 and increased re-epithelialization, whereas the difference in bacterial burden was no longer detectable on day 12 [108]. This temporal dissociation illustrates that antimicrobial and reparative effects may not develop in parallel.

AMP multifunctionality should therefore be considered as a composite biological profile rather than as an extension of antimicrobial potency alone. Antimicrobial activity, biofilm control, immunomodulation, and tissue-repair effects may differ in magnitude, dose dependence, kinetics, and cellular or microbial context and are not necessarily concordant. These activities should be evaluated through distinct experimental and clinical endpoints, and the presence of multiple mechanistic effects should not itself be interpreted as evidence of clinical efficacy.

## 5. Therapeutic Development of AMP for DFU and DFI

### 5.1. Human Endogenous and Other Naturally Occurring Peptides

Therapeutic approaches involving human endogenous AMP may include stimulation of local peptide production and direct local administration. In primary keratinocyte cultures derived from DFU biopsies, treatment with 1,25-dihydroxyvitamin D3 [1,25(OH)_2_D3] increased *DEFB4* and *CAMP* expression and enhanced secretion of hBD-2 and LL-37 [52,92]. Conditioned media from responsive cultures inhibited *E. coli* and promoted HaCaT keratinocyte migration in vitro. However, no inhibitory activity was detected against the DFU-derived *S. aureus* or *P. aeruginosa* isolates tested in the same study [52]. These findings demonstrate that endogenous AMP responses can be induced in DFU-derived keratinocytes but remain limited to an in vitro model.

Direct local administration of native human peptides has also been investigated. LL-37 has been evaluated in a topical formulation in patients with DFU, whereas hBD-2, LL-37, and Hst1 have been incorporated into hydrogels or wound dressings evaluated in diabetic animal wound models [109,110,111,112]. These studies assessed different antimicrobial, inflammatory, and wound-repair endpoints and therefore do not provide equivalent evidence of therapeutic efficacy.

A distinct subset of naturally occurring AMP comprises cryptic antimicrobial peptides, or cryptides, which are bioactive sequences embedded within larger parent proteins and exposed or released through proteolytic processing. Such peptides may originate from proteins whose principal biological functions are unrelated to antimicrobial defense [113].

AMP-IBP5 provides one example. This 22-residue amidated peptide is derived from insulin-like growth factor-binding protein 5 (IGFBP-5) through proteolytic processing involving the prohormone convertases PC1/3 and PC2. IGFBP-5 is broadly expressed in several tissues, including skin, where it is associated predominantly with keratinocytes and fibroblasts [114]. Under high-glucose conditions, AMP-IBP5 restored keratinocyte proliferation and migration and increased production of the angiogenic factors angiogenin and VEGF through pathways involving EGFR, STAT1/3, and MAPK signaling. In STZ-induced diabetic mice, it accelerated wound healing and promoted angiogenic responses and vessel formation [23]. Additional host-directed effects of AMP-IBP5 include LRP1-dependent migration and proliferation of keratinocytes and fibroblasts, increased IL-8 and VEGF production, and MrgprX2-dependent mast-cell degranulation and migration [114].

Cryptic host-defense peptides may also arise from proteolytic processing of human thrombin. TCP-25, a thrombin-derived *C*-terminal peptide, combines antibacterial activity with neutralization of pathogen-associated molecular patterns and has been incorporated into a hydrogel for local wound treatment. The TCP-25 hydrogel killed *S. aureus* and *P. aeruginosa* in vitro and in experimental mouse models of subcutaneous infection, while in a porcine partial-thickness wound model it prevented *S. aureus* infection and reduced proinflammatory cytokine levels. Proteolytic fragmentation of TCP-25 also generated bioactive fragments identical or similar to those detected in wounds in vivo [115]. Although these models were not diabetic, they provide wound-specific evidence for a cryptide-derived therapeutic approach.

Buforin-I represents another proteolytically generated AMP. It is derived from histone H2A through cleavage by pepsin isozymes in the stomach of the Asian toad and was originally identified as a 39-residue antimicrobial peptide [116]. In STZ-induced diabetic mice, Buforin-I accelerated wound closure and promoted fibroblast proliferation and collagen deposition, while under high-glucose conditions it increased HaCaT proliferation and migration and reduced apoptosis in vitro [117].

Lactoferricin B (LFcinB), a 25-residue cryptic antimicrobial peptide derived from bovine lactoferrin, provides additional diabetic-wound evidence. LFcinB promoted keratinocyte migration in vitro and in a porcine ex vivo wound model and improved wound healing in a T1DM mouse model. In diabetic wounds, treatment was also associated with changes in wound microbiota, including decreased prevalence of *S. aureus*, together with reduced oxidative stress and the M1/M2 macrophage ratio, increased angiogenesis, and collagen deposition [118].

More recently, KNR50 was identified in silico as a cationic cryptic sequence located within the *C*-terminal region of bovine αS2-casein and subsequently produced recombinantly [119]. KNR50 showed antibacterial, antibiofilm, immunomodulatory, and antioxidant activities, demonstrated anti-infective activity in a *Caenorhabditis elegans* infection model, and increased closure of scratched HaCaT monolayers in vitro. Because physiological release of KNR50 from αS2-casein was not demonstrated, its classification reflects the presence of a cryptic bioactive sequence rather than confirmed endogenous proteolytic generation.

Together, these studies identify cryptides as a heterogeneous source of AMP candidates embedded within larger proteins. Their relevance to DFU is supported mainly by cell-based and animal studies, with preclinical diabetic-wound evidence available for AMP-IBP5, Buforin-I, and LFcinB. Whether cryptide generation, stability, and activity are preserved within the protease-rich DFU microenvironment remains poorly defined.

Beyond cryptides, other naturally occurring non-human AMP provide additional molecular templates, although DFU-specific evidence remains predominantly preclinical. Bee-derived products are one source of such peptides and have been investigated in antimicrobial and wound-healing studies. In a T1DM mouse model, treatment with whole bee venom accelerated wound closure, increased collagen and β-defensin-2 expression, restored Ang-1/Nrf2/Tie2-dependent signaling, reduced oxidative stress, and improved recruitment and survival of wound macrophages [120]. Although these effects cannot be attributed to an individual venom component, they provide a biological context for the evaluation of defined bee-derived peptides. Melittin, a major peptide component of *Apis mellifera* venom, was selected in silico as a candidate AMP in a study evaluating a bee-venom-based wound dressing. The dressing inhibited *S. aureus* and *P. aeruginosa* in vitro, with ciprofloxacin used as an antibacterial comparator [121]. Jelleine-1, a natural AMP originally isolated from *A. mellifera* royal jelly, has been incorporated with 8-bromoadenosine-3′,5′-cyclic monophosphate (8Br-cAMP) into a carrier-free hydrogel that showed antibacterial activity and accelerated healing of MRSA-infected diabetic wounds in rats [122]. However, since the formulation contained both Jelleine-1 and 8Br-cAMP, the wound-healing effect cannot be attributed to Jelleine-1 alone. Jelleine-1 has also been evaluated directly for antibiofilm activity in vitro, and halogenated derivatives showed increased antibiofilm activity and proteolytic stability compared with the parent peptide [123].

Bacteriocins, ribosomally synthesized antimicrobial peptides produced by bacteria, constitute another group of naturally occurring AMP of potential interest for DFI. Nisin Z, produced by *L. lactis*, inhibited populations of *P. aeruginosa* persisters isolated from DFI but did not eradicate persisters within mature biofilms [88].

### 5.2. Synthetic and Engineered Peptides

Beyond naturally occurring AMP, synthetic or engineered peptides provide opportunities to modify antimicrobial activity, stability, selectivity, and antibiofilm properties. Rational design of peptide molecules has become a major direction of AMP development. Structural optimization may include amino acid substitution, modification of disulfide bonding, terminal modification, lipidation, and hybridization [22]. Computational approaches are also increasingly applied to peptide sequence and formulation design [124].

Representative examples include MYR-DM-ANG1-7 and CaD23. MYR-DM-ANG1-7 is a self-assembling lipopeptide composed of a myristoylated cathelicidin-DM-derived sequence linked to angiotensin-(1–7). It showed antibacterial activity against *E. coli* and *S. aureus*, inhibited biofilm formation, reduced oxidative stress and levels of the proinflammatory cytokines IL-6 and TNF-α, and promoted endothelial proliferation, migration, and angiogenic activity under high-glucose and inflammatory conditions. In an *S. aureus*-infected diabetic mouse wound model, local treatment also reduced bacterial colonization and promoted wound closure and neovascularization [125]. CaD23, a hybrid peptide designed from the middle residues of LL-37 and the *C*-terminal segment of hBD-2, rapidly killed methicillin-sensitive *S. aureus* (MSSA), MRSA, and *S. epidermidis*, and showed a low tendency toward antimicrobial resistance selection [126]. The in vivo evaluation of CaD23 was performed in a murine bacterial keratitis model rather than a diabetic wound model. Topical treatment reduced *S. aureus* burden without impairing corneal epithelial wound healing.

Additional engineered candidates have been evaluated directly in infected diabetic wound models. SR25, identified through genome mining, displayed broad-spectrum activity against Gram-positive and Gram-negative pathogens through a dual-targeting mechanism involving disruption of bacterial membrane integrity and inhibition of succinate:quinone oxidoreductase [127]. When incorporated into a hydrogel, it reduced mixed *E. coli* and MRSA infection and accelerated wound healing in diabetic mice [127]. RP557, a designed host defense peptide, suppressed bacterial growth in biofilms, reduced bacterial burden, and accelerated wound closure in MRSA-infected wounds in diabetic TALLYHO mice [128]. In contrast, RP557 did not alter wound-healing rates in uninfected TALLYHO mice, indicating that the improvement in closure observed in infected wounds was associated primarily with control of infection rather than a generalized pro-reparative effect.

PL-5/Peceleganan is a 26-residue synthetic amphipathic α-helical analogue of a cecropin A/melittin B hybrid peptide selected from a rationally designed peptide library [129]. In preclinical studies, PL-5 showed activity against Gram-positive (*S. aureus*, *S. pneumoniae*, and *S. epidermidis*) and Gram-negative (*P. aeruginosa*, *E. coli*, and *K. pneumoniae*) wound-associated pathogens. It also reduced *S. aureus* burden in a murine wound-infection model [129]. Unlike most engineered candidates discussed above, PL-5 has subsequently progressed to clinical evaluation in wound infections and DFI [130,131,132].

Synthetic and engineered AMP candidates therefore differ substantially in design strategy, antimicrobial spectrum, host–cell effects, and level of preclinical validation (Table 3). Most remain at the preclinical stage, and clinical translation will require coordinated optimization of peptide structure, stability, and delivery format.

### 5.3. Combinatorial AMP-Based Strategies

AMP-based combinations may improve antimicrobial activity while reducing the concentrations required for individual agents and may therefore be relevant to polymicrobial and biofilm-associated DFI. However, combination effects are highly context-dependent, and synergy observed against planktonic bacteria may not be preserved in biofilms or wound-like environments.

The most extensively studied strategy combines AMP with conventional antibiotics. Membrane-active peptides may increase bacterial envelope permeability and thereby facilitate access of antibiotics to intracellular targets. Chicken cathelicidins CATH-1 and CATH-3 and porcine PMAP-36 showed synergistic bactericidal activity with erythromycin against *S. aureus*, *Salmonella enteritidis*, and *E. coli* [137]. For CATH-1, the combination increased bacterial membrane permeability, delayed the emergence of erythromycin resistance in *E. coli*, and reduced hemolytic activity and mammalian-cell cytotoxicity by permitting lower concentrations of both agents [137]. LL-37 also showed synergy with colistin against MDR *E. coli*, including *mcr-1*-harboring, ESBL-producing, and carbapenemase-producing isolates [138]. LL-37 and CAMA [cecropin(1–7)-melittin A(2–9) amide] were also evaluated as adjuvants to colistin and imipenem against clinical *P. aeruginosa* isolates. LL-37 produced the more consistent reductions in antibiotic MICs, including against MDR isolates, while neither peptide showed substantial additional cytotoxicity toward human epithelial cells [139]. PL-5 also showed synergistic activity with levofloxacin against *S. aureus* both in vitro and in a murine wound-infection model [129]. Melittin-based combinations further illustrate potential antibiofilm effects: melittin with colistin or imipenem showed synergistic activity against MDR, strongly biofilm-forming *A. baumannii*, and the melittin–colistin combination markedly inhibited biofilm formation [136]. These studies provide mechanistic evidence for AMP–antibiotic combinations but were not performed in DFU-specific models.

Importantly, studies using DFI-derived isolates demonstrate that synergy cannot be assumed. HNP-1 and hBD-1 were active against MSSA and MRSA isolated from patients with DFI, but their combinations with cefotaxime showed no synergistic interaction [135]. The HNP-1/hBD-1 combination retained antimicrobial activity and performed similarly to or better than cefotaxime-containing regimens in the experimental assays, indicating that AMP activity does not necessarily translate into AMP–antibiotic synergy [135].

Engineered peptides provide further evidence of species- and model-dependent interactions. SAAP-148 (LKRVWKRVFKLLKRYWRQLKKPVR), derived from LL-37, showed synergy with teicoplanin against *S. aureus*, including biofilm-associated bacteria, whereas no synergy was observed against *S. epidermidis* [140]. SAAP-148 also showed strain-dependent synergistic or additive interactions with halicin against antimicrobial-resistant *E. coli* and *S. aureus*, including in three-dimensional (3D) human infection models [141]. These systems were not diabetic-wound models but illustrate that favorable combinations may depend on both the pathogen and the experimental environment.

AMP–AMP combinations have been evaluated more directly in DFU-relevant systems. In a collagen-based 3D DFU model, nisin A combined with pexiganan (GIGKFLKKAKKFGKAFVKILKK-NH_2_), a synthetic AMP derived from magainin 2 of *Xenopus laevis* skin, reduced the pexiganan concentration required to inhibit and eradicate both planktonic and biofilm-associated *S. aureus* and *P. aeruginosa* co-isolated from an infected DFU [142]. Incorporation of both peptides into a guar gum biogel (dual-AMP biogel) preserved antimicrobial activity, and local application eradicated *S. aureus* from the model [142]. In contrast, addition of gentamicin, clindamycin, or vancomycin to the dual-AMP biogel did not significantly improve control of *S. aureus* and *P. aeruginosa* biofilms [143]. An antagonistic interaction was observed against *S. aureus*, for which the dual-AMP biogel alone was more effective than the corresponding AMP–antibiotic combinations [143]. These findings directly demonstrate that increasing the number of antimicrobial components does not necessarily improve activity in a DFU-like biofilm environment.

Combination strategies may also couple antimicrobial activity with host-directed effects. A carrier-free hydrogel containing Jelleine-1 and 8-bromoadenosine-3′,5′-cyclic monophosphate (8Br-cAMP), a molecule that stimulates cell proliferation and growth factor production, combined local antibacterial activity with enhanced TGF-β and VEGFA production both in vitro and in vivo, leading to improved healing of MRSA-infected diabetic wounds in rats [122]. In a non-diabetic model, combining the synoeca-MP, an antimicrobial peptide of the mastoparan family isolated from the venom of the social wasp *Synoeca surinama*, with the immunomodulatory peptide IDR-1018 reduced synoeca-MP cytotoxicity without compromising its activity against *S. aureus* and promoted skin-cell proliferation, migration, pro-regenerative gene expression, and re-epithelialization in a 3D human skin equivalent [144]. This model does not establish efficacy in DFU but illustrates how peptide combinations may be designed to balance antimicrobial activity with tissue compatibility and repair.

Overall, experimental studies show that AMP-based combinations can enhance antimicrobial or antibiofilm activity, reduce exposure to individual agents, or integrate antimicrobial and reparative functions. However, these effects are species-, strain-, agent-, and model-dependent, and most evidence remains limited to in vitro and animal studies. Direct clinical evidence for combinatorial AMP therapy in DFU/DFI remains insufficient, and candidate combinations require validation in polymicrobial, biofilm-containing, and metabolically relevant diabetic-wound models before clinical efficacy can be inferred.

### 5.4. Topical and Local Delivery Approaches

Local AMP administration may reduce systemic exposure and increase peptide availability within the wound bed. This approach is particularly relevant to DFU/DFI because systemic peptide delivery is limited by rapid clearance, proteolytic degradation, and potential off-target toxicity. However, topical formulations must retain peptide activity in the presence of wound exudate, altered pH and ionic conditions, tissue ischemia, local proteases, and biofilm-associated sequestration or inactivation.

Preclinical studies have evaluated both simple topical formulations and more structured delivery systems. Niosomal gels containing HNP-1 or hBD-1 accelerated closure of MRSA-infected wounds in rats compared with untreated controls [135]. Clinical evaluation has included LL-37, pexiganan, and PL-5/Peceleganan, although the corresponding trials differ substantially in wound type, infection status, comparator, primary endpoint, and stage of development (Table 4). Clinical resolution of infection, microbiological eradication, and wound-healing outcomes have not been consistently concordant across these studies.

Clinical studies of LL-37 have primarily examined wound healing rather than treatment of established DFI. In hard-to-heal VLUs, an initial phase I/II study indicated a non-linear dose–response relationship, whereas the subsequent phase IIb study did not demonstrate significant improvement in the overall population [145,146]. Because these studies involved venous ulcers, their findings cannot be directly extrapolated to DFU. In the smaller phase II DFU trial, LL-37 increased granulation but did not significantly improve wound-area reduction, aerobic bacterial burden, or inflammatory-marker levels relative to placebo [109]. The available human evidence therefore indicates biological activity of topical LL-37 but does not establish consistent wound-closure or antimicrobial efficacy in DFU.

Pexiganan underwent clinical evaluation in multiple dedicated phase III DFI trials. The earlier studies 303 and 304 compared topical pexiganan with oral ofloxacin and produced mixed individual results, although study 304 and the pooled analysis showed comparable clinical and microbiological outcomes between treatment groups [147]. Subsequent placebo-controlled OneStep-1 and OneStep-2 trials evaluated pexiganan in addition to standardized wound care and did not demonstrate superiority over vehicle plus standardized wound care for resolution of mild DFI [148,149].

PL-5/Peceleganan has been evaluated in both heterogeneous wound-infection populations and DFI-specific studies. Phase IIb and phase III trials in heterogeneous wound infections reported higher clinical infection-response rates with PL-5 than with silver sulfadiazine, whereas culture-based bacterial clearance did not show corresponding superiority [130,131]. Because DFU represented only a small subgroup of the phase III population, that study does not provide robust evidence of DFU-specific efficacy. A subsequent randomized trial in patients with mildly to moderately infected DFU reported a higher clinical response rate with PL-5 plus standardized debridement than with placebo plus debridement, while overall microbiological eradication did not differ significantly between groups [132]. Interpretation is limited by the small sample size and a baseline imbalance in wound-infection severity, although the difference in clinical response remained after adjustment for baseline characteristics. A separate phase II trial in mild DFI (NCT06189638) is ongoing, and a phase IIIb program (ChiCTR2300071255) is evaluating PL-5 in broader wound infections [150,151].

Taken together, clinical evaluation of topical AMP has demonstrated feasibility but inconsistent efficacy across antimicrobial, infection-resolution, and wound-healing endpoints. These endpoints should therefore be assessed separately in future DFU/DFI trials.

### 5.5. Biomaterial Platforms for AMP Delivery and Wound-Targeted Therapy

**Figure 2:**
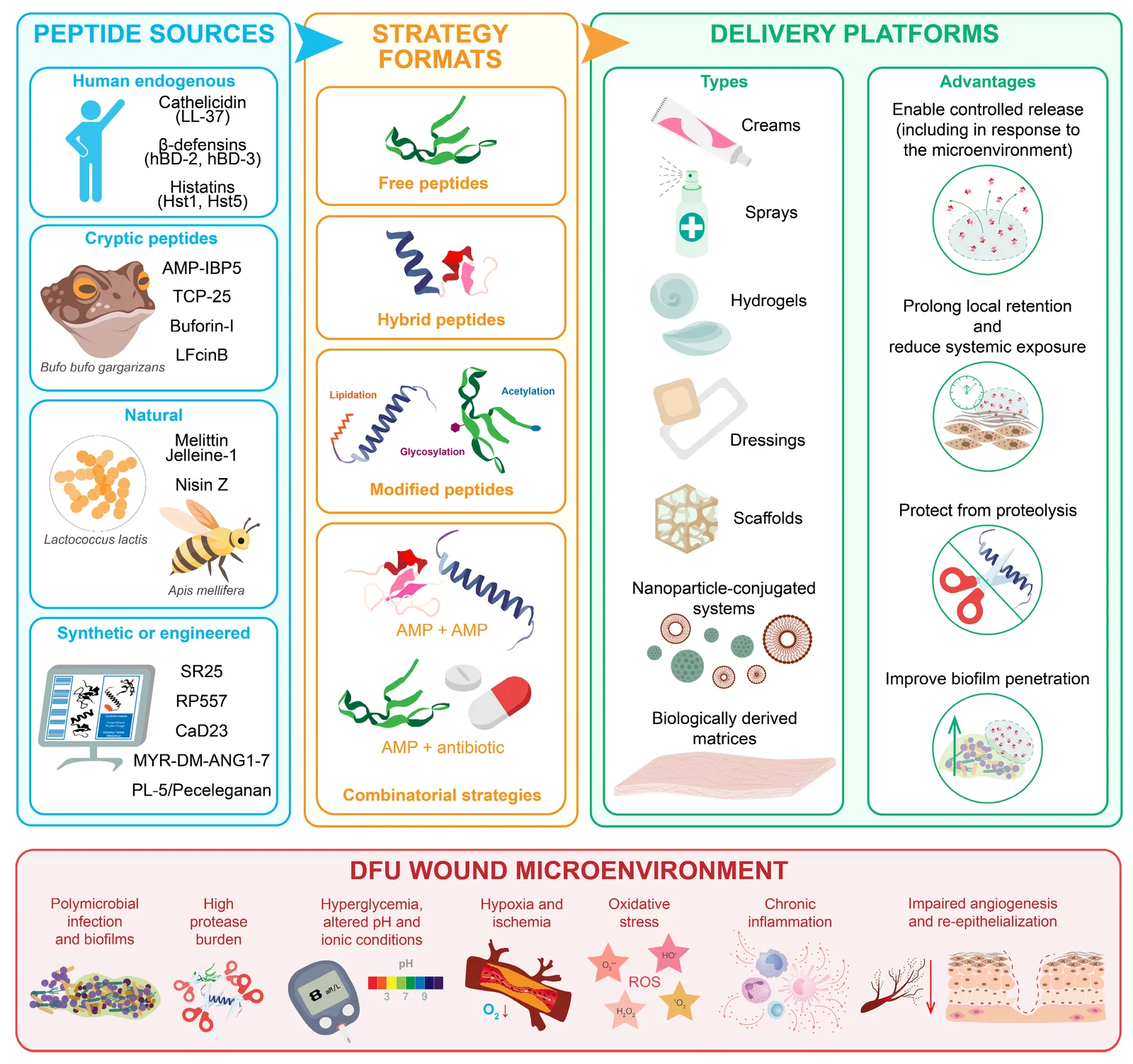
Conceptual framework for the development and local delivery of AMP-based strategies in DFU. The upper panel links peptide selection and design with subsequent therapeutic formats and delivery approaches. The lower panel depicts major characteristics of the DFU wound microenvironment that may constrain peptide stability and activity and contribute to persistent infection and impaired repair.

Biomaterial platforms can improve AMP stability, local retention, and release within the wound bed. Depending on their composition, multifunctional systems may additionally provide antioxidant, immunomodulatory, or pro-angiogenic functions. Preclinical approaches include biologically derived matrices, hydrogels, immobilized dressings, scaffolds, and peptide–nanomaterial composites. The relationship between peptide origin, molecular format, delivery strategy, and major constraints of the DFU microenvironment is summarized in Figure 2.

Biologically derived matrices differ from conventional delivery carriers because antimicrobial activity may be intrinsic to the material. Human cryopreserved viable amniotic membrane (hCVAM)-conditioned medium inhibited *P. aeruginosa*, *S. aureus*, and MRSA in vitro [152]. hBD-2 and hBD-3 were secreted by viable cells in hCVAM, and immunodepletion confirmed that both peptides contributed to its activity against *P. aeruginosa*.

Hydrogels are among the most extensively investigated platforms for local AMP delivery. Alginate hydrogels containing hBD-2 provided sustained peptide release for more than 3 days and improved wound closure, re-epithelialization, tissue remodeling, and local inflammatory parameters in an STZ-induced diabetic mouse wound model [110]. This delivery platform was subsequently evaluated in an MRSA-infected STZ-induced diabetic mouse model, where hBD-2 hydrogels accelerated wound closure, reduced MRSA burden, decreased the M1/M2 macrophage ratio, and increased proliferative and angiogenic markers [110,153].

Peptide optimization has also been combined with formulation design to preserve antimicrobial activity under wound-associated conditions. TPL6 peptide, generated from TP4 by removal of glycine-rich segments, retained α-helical structure and antimicrobial activity under high-glucose and high-salt conditions and showed lower cytotoxicity than its precursor [154]. Formulation with adjuvants as a gel restored activity under serum-rich conditions, prevented biofilm formation, and eradicated established biofilms of MDR pathogens [154]. In infected ex vivo porcine skin and diabetic mouse wound models involving MDR *S. aureus* or *C. albicans*, TPL6 gel was more effective than the corresponding antibacterial or antifungal comparator formulations [154]. These findings indicate that selected AMP formulations can target both bacterial and fungal wound pathogens. However, evidence for such dual-spectrum activity remains predominantly preclinical and has not been established clinically in DFU/DFI.

Hydrogels have also been engineered to respond to physicochemical features of the diabetic wound. The glucose- and pH-responsive QO/@PV@AB7 hydrogel contained AMP-AB7-loaded silica nanoparticles and pravastatin-loaded chitosan nanoparticles [155]. The formulation scavenged ROS, enhanced HUVEC viability and angiogenic activity, and inhibited MRSA and *E. coli* in vitro. In a rat model of MRSA-infected diabetic foot wounds, treatment reduced IL-6 and TNF-α, increased IL-10 and the angiogenesis-associated markers eNOS, VEGF, and CD31, and accelerated wound healing [155]. Because the formulation contained multiple active components, these effects cannot be attributed to AMP-AB7 alone.

An infection-responsive supramolecular hydrogel was constructed by conjugating an AMP to adamantane-modified hyaluronic acid (Ad–HA) through a cyclic peptide linker containing MMP- and ROS-cleavable sequences [156]. AMP release occurred only when the hydrogel was exposed simultaneously to MMPs and ROS. The Ad–HA–AMP conjugate showed increased serum stability and longer-lasting antimicrobial activity than the native AMP. In infected diabetic mouse wounds, the AMP-tethered hydrogel accelerated wound healing [156].

Externally activated systems provide another mechanism for controlling antimicrobial activity. In the sonodynamic PPCN@Pt-AMPs/HGel dressing, the biofilm-targeting peptide HHC-36 was incorporated into a composite system containing the nanosonosensitizer PCN-224 and an oxygen-generating Pt nanozyme [20]. Under low-intensity ultrasound, the complete formulation inhibited newly formed biofilms and disrupted mature biofilms in vitro. In rats with infected diabetic wounds, ultrasound-assisted treatment accelerated wound closure and was associated with increased granulation tissue formation, angiogenesis, and type III collagen deposition [20].

Surface immobilization of AMP-containing nanoparticles has also been investigated for wound-dressing applications. A polyurethane-based dressing containing surface-immobilized LL37-conjugated gold nanoparticles (PU-adhesive-LL37NP) showed antibacterial activity against Gram-positive and Gram-negative bacteria in human serum and did not induce detectable bacterial resistance during 16 sequential antimicrobial test cycles [111]. In an uninfected full-thickness wound model in db/db mice, the dressing enhanced re-epithelialization and reduced macrophage infiltration relative to the polyurethane control [111]. Moreover, lower TNF-α and IL-6 expression was observed after 6 days of treatment.

Matrix-based delivery has also been evaluated for Hst1, with the reported effects being predominantly reparative rather than antimicrobial. Hst1 incorporated into a conductive gelatin/chitosan hydrogel containing polypyrrole-based nanoparticles accelerated healing of full-thickness wounds in db/db diabetic mice and was associated with reduced TNF-α and increased CD31 and α-SMA expression [112]. In a separate study, fusion of Hst1 with a collagen-binding domain enabled its immobilization on an acellular dermal matrix and sustained local release. The resulting C-Hst1/ADM scaffold promoted adhesion, migration, and angiogenic activity of vascular endothelial cells in vitro and increased angiogenesis and extracellular collagen accumulation while reducing scar width in diabetic wounds in rats [157].

A two-step delivery strategy was evaluated using 3D radially aligned nanofiber scaffolds containing W379, an LL-37-mimetic AMP, with or without platelet-derived growth factor-BB (PDGF-BB) [158]. Initial treatment used rapidly released free W379 to reduce the established *S. aureus* biofilm, followed by scaffolds providing sustained W379 release from microspheres. PDGF-BB was additionally incorporated into the combined-treatment scaffold. In an *S. aureus* biofilm-infected STZ-induced diabetic mouse wound model, W379-containing scaffolds markedly reduced bacterial burden, whereas the W379/PDGF-BB formulation produced the greatest effects on granulation tissue formation, neovascularization, re-epithelialization, and wound closure [158].

Overall, biomaterial platforms can improve local AMP stability, retention, and release while incorporating additional wound-directed functions. However, the evidence remains limited to in vitro, ex vivo, and animal studies, and the AMP-containing biomaterial systems discussed here have not yet been clinically evaluated specifically in patients with DFU.

## 6. Translational Challenges and Future Directions

Despite the growing number of AMP candidates and delivery platforms, translation of AMP-based therapies to DFU and DFI treatment remains limited by peptide instability within the wound environment, host-tissue compatibility, insufficiently predictive preclinical models, and challenges in clinical trial design, manufacturing, and regulatory development.

### 6.1. AMP Stability and Activity in the Diabetic Wound Environment

AMP activity in DFU depends on preservation of peptide integrity and function within the chronic wound environment. Proteases, wound exudate, increased ionic strength, serum proteins, pH variation, and biofilm-associated components can reduce peptide availability through enzymatic degradation, binding, and other physicochemical interactions. Activity measured under simplified in vitro conditions may therefore overestimate performance in the wound bed.

LL-37 provides a well-characterized example of these effects. The *S. aureus* metalloprotease aureolysin cleaves LL-37 in a time- and concentration-dependent manner, resulting in loss of antibacterial activity. In contrast, V8 protease (glutamyl endopeptidase) predominantly cleaves the Glu16–Phe17 bond, generating the *C*-terminal fragment LL-17-37, which is resistant to further V8-mediated degradation and retains antibacterial activity [159,160]. LL-37 is also susceptible to cleavage by several *C. albicans* secreted aspartic proteases [159]. Progressive proteolytic processing is associated with loss of antifungal activity, although the major intermediate LL-25 retains antifungal activity while lacking several immunomodulatory properties of full-length LL-37. In biological fluids, LL-37 activity can additionally be inhibited by interactions with polyelectrolytes and other macromolecules, including glycosaminoglycans, collagen, and actin. It is also influenced by ionic composition and pH [159]. These observations indicate that AMP activity should be assessed under wound-relevant physicochemical conditions in addition to standard antimicrobial assays.

Strategies used to improve proteolytic stability and peptide persistence include partial or complete substitution with D-amino acids or other non-natural residues, sequence optimization, peptide cyclization or stapling, peptidomimetic backbone design, lipidation, PEGylation, and terminal modifications [22,161]. In Trp/Arg-rich AMP analogues, *N*-terminal acetylation increased serum stability but reduced antimicrobial activity, whereas peptide cyclization improved proteolytic stability with variable effects on antimicrobial potency [162]. The all-D-amino acid form of LL-37 was resistant to trypsin degradation while retaining antimicrobial and antibiofilm activity against *P. aeruginosa* [163]. For SAMP-A4, *N*-terminal fatty-acid conjugation improved stability in serum and trypsin more effectively than the glycosylation and PEGylation strategies examined and was accompanied by increased antimicrobial activity against MDR bacteria and *Candida* spp. [164]. More recently, terminally capped and all-D synthetic AMP analogues retained antimicrobial activity under protease-rich, high-salt, and serum-rich conditions modelling selected features of chronic wound exudate, while the all-D analogue also showed complete resistance to trypsin degradation and retained antibiofilm activity [165]. These findings indicate that increased proteolytic stability can be achieved without necessarily compromising antimicrobial or antibiofilm activity, but their functional effects depend on peptide sequence, length, and modification strategy.

Formulation design provides a complementary approach to maintaining local peptide exposure and activity under wound-associated conditions. Sustained-release hydrogels and other local delivery systems can prolong peptide retention within diabetic wounds [110,153], while formulation strategies have also been used to preserve AMP activity in serum-rich or otherwise inhibitory environments [154]. Computationally guided biomaterial design has also been investigated. An AI-designed AMP hydrogel exhibited high antibacterial activity against MRSA and *E. coli* in vitro and reduced MRSA burden in a non-diabetic dynamic rat wound model [124]. Such approaches remain preclinical and require validation under the combined biochemical and microbiological conditions characteristic of DFU.

Cell-based systems have also been investigated as AMP expression and delivery platforms. For example, genetically modified Wharton’s jelly mesenchymal stem cells expressing SE-33, a truncated retro-analogue of LL-37, reduced bacterial burden and inflammation in an *S. aureus*-induced pneumonia model in mice [166,167]. Because these systems have not been evaluated in diabetic wound models, their relevance to topical DFU treatment remains uncertain and would require additional assessment of cell persistence, containment, dosing, and local safety.

### 6.2. Safety and Selectivity: Balancing Antimicrobial Activity with Tissue Compatibility

AMP development requires preservation of antimicrobial activity without unacceptable toxicity toward host tissues. Membrane-targeting peptides may cause cytotoxicity or hemolysis, while immunomodulatory activity can vary with peptide concentration, cell type, and inflammatory context.

LL-37 illustrates the need to control local exposure. In addition to its reparative and pro-angiogenic functions, LL-37 has been associated with proliferative effects in malignant skin cells. Increased LL-37 expression has been reported in melanoma and squamous cell carcinoma, and exogenous LL-37 promoted proliferation, migration, and invasion of melanoma cells in vitro [159]. These observations do not establish a carcinogenic effect of topical LL-37 treatment, but support control of peptide concentration, duration of exposure, and tissue distribution.

Sequence optimization, peptide combinations, and localized delivery can improve the balance between antimicrobial activity and host–cell compatibility. In human fibroblasts, keratinocytes, and melanocytes, IDR-1018 reduced the cytotoxic effects of synoeca-MP without loss of its antibacterial activity against *S. aureus.* The combination also increased cell proliferation and migration and enhanced re-epithelialization in a 3D human skin equivalent model [144]. TPL6 similarly showed lower cytotoxicity than its TP4 precursor while retaining broad antimicrobial activity [154]. Safety assessment should therefore include not only conventional cytotoxicity and hemolysis assays but also effects on wound-relevant epithelial, stromal, endothelial, and immune cells at therapeutically relevant exposure levels.

### 6.3. From Simplified Assays to DFU-Relevant Preclinical Models

The predictive value of many AMP studies is limited by the use of planktonic cultures, single-species biofilms, and simplified in vitro cell models. These systems do not reproduce the combined effects of hyperglycemia, impaired perfusion, oxidative and proteolytic stress, altered host–cell responses, and polymicrobial biofilms characteristic of diabetic wounds. AMP performance under these conditions depends on both microbial susceptibility and surrounding tissue environment.

More advanced in vitro 3D and ex vivo models can provide intermediate evidence between conventional antimicrobial assays and animal studies. In a collagen-based 3D DFU model containing *S. aureus* and *P. aeruginosa* clinical isolates, combined pexiganan and nisin reduced the peptide concentrations required for antimicrobial activity, and the dual-AMP biogel eradicated *S. aureus* but showed more limited activity against P. aeruginosa [142]. A subsequent study using the same general model found no consistent improvement when the dual-AMP biogel was combined with gentamicin, clindamycin, or vancomycin and identified antagonistic interactions against *S. aureus* under some conditions [143]. In a 3D human skin equivalent, synoeca-MP/IDR-1018 retained antimicrobial activity while increasing re-epithelialization and expression of genes associated with regeneration [144]. TPL6 gel was evaluated in infected ex vivo porcine skin and diabetic mouse wounds involving MDR *S. aureus* or *C. albicans*, providing tissue-level and animal evidence under drug-resistant bacterial and fungal infection conditions [154].

Future models should combine polymicrobial biofilms with major host abnormalities relevant to DFU, including hyperglycemia, impaired cell migration, inflammatory dysregulation, defective angiogenesis, tissue hypoxia or ischemia, and increased oxidative and proteolytic stress. This is particularly important for responsive and multicomponent platforms whose activity depends on local wound conditions, including glucose- and pH-responsive hydrogels [155], MMP/ROS-dependent delivery systems [156], ultrasound-activated antibiofilm platforms [168], and staged scaffolds combining early biofilm control with subsequent support of tissue repair [158]. Thus, model complexity alone is insufficient. Experimental systems should reproduce the specific microbial and host constraints that determine therapeutic performance in DFU.

### 6.4. Clinical Translation: Endpoints, Manufacturing, and Regulatory Considerations

Clinical evidence for AMP treatment of DFU and DFI remains limited and heterogeneous. Studies of pexiganan, LL-37, and Peceleganan have shown discordance between clinical, microbiological, and wound-healing outcomes. Pexiganan reached phase III evaluation in infected DFU without establishing consistent clinical benefit, while LL-37 and Peceleganan studies demonstrated that improvement in one outcome domain may occur without corresponding improvement in another. Infection-related and wound-healing outcomes should therefore be evaluated separately.

The primary endpoint should correspond to the intended therapeutic indication. For antibacterial DFI drug trials without bone or joint involvement, current FDA guidance recommends a primary endpoint based on resolution or improvement of the signs and symptoms of infection to the extent that no further antibacterial therapy is required [169]. Rescue therapy, unplanned surgical debridement, amputation, or death are considered treatment failures. DFI is likewise defined clinically in the IWGDF/IDSA guideline, which recommends against quantitative microbial analysis for diagnosis [170]. For products intended primarily to promote wound healing, FDA guidance for chronic wound products identifies complete wound closure as an objective and clinically meaningful endpoint and does not consider partial wound healing alone sufficient as a phase III primary endpoint [171]. The core outcome set for diabetes-related foot ulcer studies additionally includes wound healing, time to healing, new or recurrent ulceration, infection, major and minor amputation, health-related quality of life, and mortality [172]. For multifunctional AMP systems, infection-related and wound-healing outcomes should be prespecified and analyzed separately. Improvement in one domain does not establish efficacy in the other.

Trial design should also account for factors that influence DFI outcome independently of the investigational treatment. Current regulatory guidance identifies infection severity, vascular insufficiency, glycemic control, and previous antibacterial therapy as relevant variables in trial design and analysis and treats concomitant osteomyelitis separately because of differences in management and outcome assessment [169]. The IWGDF/IDSA guideline emphasizes wound cleansing and debridement, pressure off-loading, vascular assessment and revascularization when indicated, and metabolic control as components of DFI management [170]. Background care should therefore be standardized and reported across treatment groups.

Manufacturing requirements depend on both the peptide and the formulation. Current industry practices for synthetic peptide control strategies include identity, purity, assay, impurity testing and, where applicable, bioassay and comparability assessment, with differences between drug substance and drug product and across stages of development [173]. Peptide-related impurities require specific analytical assessment. Their characterization cannot be based directly on conventional small-molecule approaches, and aggregates and adducts represent relevant impurity classes [174]. Nanomaterial-based formulations introduce additional manufacturing and quality-control requirements, including characterization of physicochemical properties, peptide loading, in vitro release, excipients, and product stability [175].

Clinical development of AMP products for DFU and DFI should therefore define the intended treatment claim, the corresponding primary endpoint, background standard of care, and formulation-specific control strategy. Antimicrobial activity alone does not establish either clinical resolution of infection or improved wound healing.

## 7. Conclusions

DFU and DFI involve interacting microbial and host factors, including polymicrobial biofilms, antimicrobial resistance, inflammation, ischemia, and impaired tissue repair. Preclinical evidence supports further investigation of AMP and local delivery systems with antimicrobial, antibiofilm, immunomodulatory, or pro-reparative activity. However, clinical evidence specifically in DFU and DFI remains limited and inconsistent, and AMP-containing biomaterial systems have not yet demonstrated clinical efficacy in these settings.

Future studies should evaluate the integration of AMP engineering with advanced local delivery platforms, including responsive hydrogels, nanoparticle-based formulations, and other wound-adapted biomaterials, as well as with conventional antibiotics, while separately assessing peptide stability, antimicrobial activity, tissue compatibility, and clinically relevant outcomes.

Important research gaps remain. These include randomized controlled trials specifically evaluating AMP-containing biomaterials in DFU or DFI, standardized clinical endpoints that distinguish infection control from wound healing, cost-effectiveness analyses, and preclinical models that simultaneously incorporate polymicrobial biofilms, hyperglycemia, ischemia or hypoxia, and impaired host repair. Further development should focus on candidates that retain activity under DFU-relevant conditions and demonstrate reproducible safety and clinically meaningful benefit in appropriately designed studies.

## Figures and Tables

**Table 1:** Geographic heterogeneity of microbial composition in DFI.

Region	Dominant Microbial Profile	Polymicrobial Infection	Anaerobic or Fungal Component	Key Reported Feature	Reference
Central Russia	Gram-positive flora predominated over Gram-negative flora (76.3% vs. 23.7%). The main Gram-positive isolates were *S. aureus* (58.8%) and *E. faecalis* (17.5%), whereas the leading Gram-negative bacteria included *Enterobacter cloacae* (10.6%), *A. baumannii* (4.4%), *E. coli* (4.4%).	DFU microbiota was monomicrobial in 79.2% of cases. Mixed communities were usually two- or three-species associations, most often involving both Gram-positive and Gram-negative organisms. Of all mixed associations, 42% consisted of *S. aureus* and *E. faecalis*.	Not reported	Predominantly Gram-positive, staphylococcal-dominant, and largely monomicrobial profile.	[62]
India	Gram-negative bacteria predominated, including *P. aeruginosa* (15.3–21.7%), *K. pneumoniae* (17.6%), *E. coli* (14.9–20%), and *P. mirabilis* (14.9%), whereas *S. aureus* accounted for 2.7–25% of isolates and remained the leading Gram-positive pathogen.	Polymicrobial infections were reported in up to 43.1% of DFU cases, although one study described an entirely monomicrobial series.	Not reported	Gram-negative-predominant profile with substantial between-study heterogeneity in polymicrobial infection.	[63,64,65,66]
Bangladesh	Mixed but Gram-negative-predominant profile, with frequent *Pseudomonas* spp. (22%), *Bacillus* spp. (12%), *Enterobacter* spp. (22%), *Staphylococcus* spp. (13%), *Acinetobacter* spp. (10%), *Enterococcus* spp. (9%) and *Klebsiella* spp. (8%).	DFI cases were polymicrobial.	Not reported	Predominance of Gram-negative organisms.	[61]
Egypt	Staphylococci predominated, including *S. aureus* (37.5%), *S. epidermidis* (34.3%) and *S. haemolyticus* (23.4%).	Polymicrobial infection was identified in 54 of 100 infected DFU.	Not reported	Staphylococcal-dominant profile with frequent co-isolation of multiple staphylococcal species (17.1% of samples).	[68]
Lebanon	Aerobic Gram-negative bacilli predominated over Gram-positive cocci (55% vs. 39%). Frequent isolates included *E. coli* (15%), *Enterococcus* spp. (14%), *P. aeruginosa* (11%), and *S. aureus* (9%).	Polymicrobial infection was identified in 54% of hospitalized patients with DFU.	Anaerobes accounted for 1% of isolates. *Candida* spp. accounted for 5% of isolates	Gram-negative-predominant profile with substantial polymicrobial burden and limited anaerobic and fungal detection by routine culture.	[67]
Saudi Arabia	Gram-negative bacteria were most common (65.7%) with *E. coli* (12%), *K. pneumoniae* (9.7%), and *P.* *aeruginosa* (8.4%) predominating, followed by *P. mirabilis* and *Citrobacter freundii*. Gram-positive bacteria accounted for 27.8%, with *S. aureus* (13.2%), *E. faecalis* and *S. epidermidis* being the most frequent.	Polymicrobial infection was detected in 54% of patients with DFU.	Anaerobic bacteria represented 6.5% of isolates, most commonly *Peptostreptococcus anaerobius* (2.3%) and *Bacteroides* spp.	Polymicrobial, Gram-negative-predominant profile with a detectable anaerobic component and a high burden of biofilm-forming isolates.	[69]

**Table 2:** Biofilm-associated tolerance, antimicrobial resistance mechanisms, and clinical implications in patients with DFU across different geographic regions.

Region	Biofilm Burden and Tolerance Evidence	Associated Pathogens	MDR Association and Phenotypes	Antibiotic Classes Compromised	Clinical Implication	Reference
Central Russia	Not reported	*S. aureus*, *E. faecalis*, *Enterobacteriaceae* and *A. baumannii* complex	High prevalence of oxacillin-resistant *S. aureus* (47.9%), marked resistance of *E. faecalis* to ampicillin and fluoroquinolones; limited active options against *Enterobacteriaceae* and *A. baumannii* complex	Anti-staphylococcal penicillins (oxacillin); Penicillins (ampicillin); Fluoroquinolones	Even in a Gram-positive-dominant cohort, resistance burden substantially constrains therapy	[62]
India	Biofilm-forming strains were detected in 42–71% of isolates. *P. aeruginosa* was often a major biofilm former	*P. aeruginosa*, *E. coli*, *P. mirabilis*, *K. pneumoniae*, other *Enterobacteriaceae*, *S. aureus*	Overall MDR burden reached 67%; particularly high in *E. coli* (97.3%), *P. mirabilis* (90.5%), and *S. aureus* (83.9%). ESBL phenotype in 31.1–53% of Gram-negative isolates; MRSA in 41–51.8% of *S. aureus*; vancomycin-resistant *Enterococcus* spp. in 19–33.3%; ESBL genes dominated by *blaCTX-M* (38.1%), followed by *blaSHV* (19.0%), and *blaTEM* (6.7%)	Penicillins (ampicillin, piperacillin); Anti-staphylococcal penicillins (methicillin, oxacillin); β-lactam/β-lactamase inhibitor (amoxicillin-clavulanic acid) Fluoroquinolones; Cephalosporins (ceftazidime, ceftriaxone, cefotaxime, cefepime); Glycopeptides (vancomycin); Macrolides (erythromycin)	Biofilm formation was associated with MDR, polymicrobial infection, and amputation in this cohort	[63,64,65,66]
Bangladesh	Not reported	*Pseudomonas* spp., *Enterobacter* spp., *Klebsiella* spp., *Acinetobacter* spp., *Bacillus* spp., *Staphylococcus* spp.	*Staphylococcus* spp. showed 100% resistance to aztreonam and 67% resistance to penicillin; *Pseudomonas* spp. were resistant to imipenem (33%), cephalosporins (72%), and aztreonam (78%); *Acinetobacter* spp. showed 86% resistance to penicillin and cephalosporins	Penicillins (penicillin); Cephalosporins (cefuroxime, ceftriaxone, ceftazidime); Monobactams (aztreonam); Carbapenems (imipenem)	Elevated serum CRP correlated with resistance burden. DFI cases with CRP > 100 mg/L were associated with *Bacillus* spp. and *Pseudomonas* spp. and broader MDR	[61]
Egypt	All staphylococcal isolates were biofilm formers of variable intensity. Stronger biofilm formation was associated with higher burden of biofilm-related genes, such as *icaD*, *spa*, and *bap*	*S. aureus*, *S. epidermidis*, *S. haemolyticus*	High MDR burden among staphylococci (78.1%). Methicillin resistance was frequent, with cefoxitin resistance and *mecA* positivity in 68.7% of isolates. Marked clonal heterogeneity in *S. aureus* (17 *spa* types)	Anti-staphylococcal penicillins (oxacillin); β-lactam/β-lactamase inhibitor (amoxicillin-clavulanic acid); Cephalosporins (cefoxitin)	In staphylococcal-dominant DFI, biofilm-forming capacity, methicillin resistance, and strain heterogeneity may jointly contribute to persistence and therapeutic complexity	[68]
Lebanon	Not reported	*S. aureus* *Enterobacteriaceae*	Approximately 50% of *S. aureus* isolates were MRSA. Among Enterobacteriaceae, 37% were fluoroquinolone-resistant, 25% were ESBL producers, and 2% were carbapenem-resistant	Anti-staphylococcal penicillins (methicillin, oxacillin); Fluoroquinolones; Carbapenems; Cephalosporins	Antibiotic resistance was significantly associated with prior antibiotic exposure, underscoring hospital-associated selection pressure in DFI	[67]
Saudi Arabia	61.5% of isolates showed biofilm-forming capacity	*S. aureus*, *S. epidermidis*, *E. coli*, *P. aeruginosa*	Overall MDR prevalence reached 69.5%, higher in Gram-negative than Gram-positive bacteria (75.9% vs. 54.6%). Among Gram-negative organisms, 40% were ESBL producers, 10.8% were carbapenemase producers, and 16.7% were AmpC β-lactamase producers. MRSA isolates (81.5%) carried *mecA*. ESBL-producing organisms carried *blaCTX-M* (27.2%). Carbapenem-resistant isolates (25.6%) often harbored *blaNDM* (69.3%). Among staphylococci, 65.8% of *S. aureus* were MRSA and 50% of *S. epidermidis* were MRSE; vancomycin resistance was also detected in a subset of *S. aureus* and *S. epidermidis*	Penicillins (ampicillin, penicillin); Anti-staphylococcal penicillins (methicillin, oxacillin); β-lactam/β-lactamase inhibitor (amoxicillin-clavulanic acid); Cephalosporins (ceftazidime, ceftriaxone, cefotaxime, cefoxitin); Fluoroquinolones (ciprofloxacin, levofloxacin); Carbapenems (imipenem); Lincosamides (clindamycin); Macrolides (erythromycin); Folate pathway inhibitors (trimethoprim-sulfamethoxazole)	Biofilm formation increased the risk of MDR infection nearly 5-fold, while polymicrobial infection increased it more than 7-fold	[69]
Tunisia	Biofilm production was generally low. One *lasR*-truncated strain displayed a chronic infection phenotype with high biofilm formation.	*P. aeruginosa*	25% of isolates were MDR, and three of four MDR isolates were carbapenem-resistant. The population was genetically heterogeneous and included virulence-rich strains	Aminoglycosides (netilmicin); Cephalosporins (cefepime); Carbapenems	Genetically heterogeneous *P. aeruginosa* population including MDR and carbapenem-resistant isolates	[70]
Israel	Severe cases of DFI showed enrichment of genes related to biofilm production, quorum sensing, and virulence	*P. aeruginosa*, *E. coli* and *Vibrio cholerae*.	43% of cultured isolates exhibited at least one recognized resistance mechanism, including MRSA, AmpC β-lactamase, ESBL, MDR Gram-negative bacilli (*P. aeruginosa*) and carbapenem-resistant *Enterobacteriaceae*. Metagenomic analysis also revealed enrichment of genes related to β-lactam and vancomycin resistance pathways	Penicillins/β-lactams; Glycopeptides (vancomycin); Carbapenems	Amputation was associated with increased abundance of genes related to biofilm production, antibiotic resistance and bacterial virulence factors. *Bacteroides* spp. were associated with amputation	[60]

**Table 3:** Representative antimicrobial peptides and AMP-derived candidates relevant to wound treatment and infection control. Peptide sequences were obtained from the cited primary studies and, where available, cross-checked against CAMPR4 (Collection of Anti-Microbial Peptides) (https://camp.bicnirrh.res.in/index.php, accessed on 20 October 2025) and APD6 Antimicrobial Peptide Database (https://aps.unmc.edu/, accessed on 20 October 2025) databases [133,134].

Peptide	Sequence	Origin or Design	Antimicrobial and Antibiofilm Activity	Immunomodulatory and Wound-Healing Effects	Stage of Development	References
Human endogenous peptides						
LL-37	LLGDFFRKSKEKIGKEFKRIVQRIKDFLRNLVPRTES	Human cathelicidin	Broad activity against Gram-positive and Gram-negative bacteria. Antifungal activity against selected fungi, including *C. auris.* Activity against *S. aureus* biofilms.	Modulates inflammatory responses. Promotes angiogenesis and re-epithelialization. Promotes diabetic wound repair through TFEB-dependent autophagy.	Clinical	[81,85,87,93,95,99,109]
HNP-1	ACYCRIPACIAGERRYGTCIYQGRLWAFCC	Human α-defensin (neutrophil peptide)	Antimicrobial activity is strain- and species-dependent. Activity against DFI-derived MSSA and MRSA isolates. Antibiofilm activity was not evaluated in the DFI-focused study.	Accelerated closure of MRSA-infected wounds in a rat model when delivered in a niosomal formulation.	Preclinical	[77,135]
hBD-1	DHYNCVSSGGQCLYSACPIFTKIQGTCYRGKAKCCK	Human β-defensins	Activity against DFI-derived MSSA and MRSA isolates. Antibiofilm activity was not evaluated in the DFI-focused study.	Promotes angiogenin secretion by dermal fibroblasts. Niosomal hBD-1 accelerated closure of MRSA-infected wounds in a rat model.	Preclinical	[101,135]
hBD-2	GIGDPVTCLKSGAICHPVFCPRRYKQIGTCGLPGTKCCKKP		Broad antibacterial activity. hBD-2 hydrogel reduced wound microbial load in a diabetic mouse model. Antibiofilm activity was not established in the DFU/DFI-focused studies.	Promotes fibroblast migration and angiogenin secretion. Supports skin-barrier function. Hydrogel delivery in diabetic wounds reduced inflammatory and oxidative responses and increased re-epithelialization, neovascularization, and collagen deposition.	Preclinical	[77,101,103,110]
hBD-3	GIINTLQKYYCRVRGGRCAVLSCLPKEEQIGKCSTRGRKCCRRKK		Broad antibacterial activity, including activity against staphylococci. Inhibits formation and viability of antibiotic-resistant *Staphylococcus* biofilms.	Induces keratinocyte migration, proliferation, and cytokine and chemokine responses. Supports angiogenesis and re-epithelialization in infected diabetic wounds.	Preclinical	[48,49,76,78,89,101,108]
Hst1	DSHEKRHHGYRRKFHEKHHSHREFPFYGDYGSNYLYDN	Human salivary histatins	Anticandidal activity, although weaker than that of Hst5. Antibiofilm activity in DFU/DFI-relevant models has not been established.	Promotes keratinocyte and fibroblast migration, endothelial responses, angiogenesis, and protection from oxidative and high-glucose injury. Promotes repair in diabetic wound models.	Preclinical	[82,105,106,112]
Hst5	DSHAKRHHGYKRKFHEKHHSHRGY		Predominantly antifungal activity against *Candida* spp., *C. neoformans*, and *A. fumigatus*. Inhibits biofilm formation and reduces biofilm metabolic activity in *C. albicans.*	Promotes epithelial migration and wound closure in non-diabetic wound models. Direct diabetic wound-healing evidence has not been established.	Preclinical	[82,83,84,90,107]
Cryptic antimicrobial peptides						
AMP-IBP5	AVYLPNCDRKGFYKRKQCKPSR-NH_2_	Peptide derived from IGFBP-5	Activity against multiple bacterial and fungal species.	Restores high-glucose-impaired keratinocyte proliferation and migration and angiogenin/VEGF production. Promotes angiogenesis and accelerates wound healing in STZ-induced diabetic mice.	Preclinical	[23,114]
TCP-25	GKYGFYTHVFRLKKWIQKVIDQFGE	Human thrombin-derived peptide	Activity against *S. aureus* and *P. aeruginosa* in vitro and in mouse infection models. Prevents *S. aureus* infection in a porcine partial-thickness wound model. Antibiofilm activity was not assessed.	Neutralizes bacterial PAMPs, including LPS, suppresses downstream inflammatory signaling, and reduces proinflammatory cytokine responses in non-diabetic infection and wound models.	Preclinical	[115]
Buforin-I	AGRGKQGGKVRAKAKTRSSRAGLQFPVGRVHRLLRKGNY	Histone H2A-derived peptide from the stomach tissue of *Bufo bufo gargarizans*	Broad-spectrum antimicrobial activity in vitro. Antibiofilm activity was not evaluated in the diabetic-wound study.	Accelerates wound closure in STZ-induced diabetic mice, with increased fibroblast proliferation and collagen deposition and reduced oxidative stress. Promotes HaCaT proliferation and migration and reduces apoptosis under high-glucose conditions.	Preclinical	[117]
LFcinB	FKCRRWQWRMKKLGAPSITCVRRAF	Peptide derived from bovine lactoferrin	Broad antimicrobial activity. Treatment of diabetic wounds altered the cultivable microbiota, including lower *S. aureus* prevalence. Antibiofilm activity was not assessed in the diabetic-wound study.	Promotes keratinocyte migration. Reduces oxidative stress and the M1/M2 macrophage ratio. Increases angiogenesis and collagen deposition and accelerates diabetic wound healing.	Preclinical	[118]
KNR50	KNRLNFLKKISQRYQKFALPQYLKTVYQHQKAMKPWIQPKTKVIPYVRYL	Peptide derived from bovine αS2-casein. Identified in silico and produced recombinantly	Broad antibacterial activity, including Gram-positive and Gram-negative bacteria. Inhibits biofilm attachment and formation in *S. enterica* and *L. monocytogenes*, with weaker activity against preformed biofilms.	Immunomodulatory and antioxidant activity. Increases closure of scratched HaCaT monolayers and wound-repair gene expression in vitro. No diabetic-wound model has been reported.	Preclinical	[119]
Naturally occurring peptides						
Melittin	GIGAVLKVLTTGLPALISWIKRKRQQ	Major peptide component of *A. mellifera* venom	Broad antibacterial activity, including activity against *S. aureus* and *P. aeruginosa*. Antibiofilm activity has been demonstrated in vitro against bacterial biofilms.	Peptide-specific reparative effects in diabetic wounds remain insufficiently defined. Cytolytic activity limits therapeutic exposure and requires formulation or dose optimization.	Preclinical	[121,136]
Jelleine-1	PFKLSLHL	Peptide derived from *A. mellifera* royal jelly	Activity against Gram-positive and Gram-negative bacteria and fungi. Antibiofilm activity has been demonstrated in vitro. In the diabetic-wound study, Jelleine-1 was incorporated into an MRSA-active hydrogel with 8Br-cAMP.	In combination with 8Br-cAMP, enhances VEGF/TGF-β-associated responses and accelerates healing of MRSA-infected diabetic wounds. The reparative effects cannot be attributed to Jelleine-1 alone.	Preclinical	[122,123]
Nisin Z	ITSISLCTPGCKTGALMGCNMKTATCNCSIHVSK	Bacteriocin produced by *Lactococcus lactis* subsp. *lactis*	Activity against *P. aeruginosa* DFI-derived persisters. Inhibits planktonic persisters but does not eradicate persister cells within mature biofilms.	No direct immunomodulatory or wound-healing effects were evaluated in the cited DFI study.	Preclinical	[88]
Engineered, hybrid, and synthetic peptides						
MYR-DM-ANG1-7	Not provided	Engineered lipopeptide composed of *N*-myristoylated cathelicidin-DM (*Duttaphrynus melanostictus*) linked to angiotensin-(1–7) via a GGG spacer	Activity against *E. coli* and *S. aureus*. Inhibits biofilm formation.	Reduces oxidative stress and IL-6 and TNF-α levels under high-glucose/inflammatory conditions and promotes endothelial proliferation, migration, and angiogenic responses in vitro. In infected diabetic mouse wounds, increases collagen deposition and neovascularization and accelerates wound closure.	Preclinical	[125]
CaD23	KRIVQRIKDWLRKLCKKW	Hybrid peptide derived from LL-37 and hBD-2 motifs	Rapid killing of MSSA, MRSA, and *S. epidermidis*. Moderate activity against *P. aeruginosa*. No resistance development was detected during 10 serial passages of *S. aureus*. Antibiofilm activity was not evaluated in the present study.	At concentrations up to 0.05%, did not delay re-epithelialization in a non-diabetic murine corneal wound model. Direct pro-reparative or immunomodulatory activity was not established.	Preclinical	[126]
SR25	SAGFVSLVCRGCRWRRRRRRLRIVR	Synthetic peptide identified through genome mining of the bovine enteric actinomycete *Nonomuraea jilinensis* sp. nov.	Broad activity against Gram-positive and Gram-negative bacteria, including drug-resistant strains. Inhibits *E. coli* biofilm formation, reduces established biofilm, and eliminates persister cells in vitro.	Hydrogel-delivered SR25 reduces mixed *E. coli*/MRSA burden and accelerates closure, re-epithelialization, and collagen deposition in infected diabetic wounds.	Preclinical	[127]
RP557	RFCWKVCYKGICFKKCK-NH_2_	Designed host defense peptide	Activity against MRSA. Inhibits bacterial growth within MRSA biofilm-containing infected diabetic wounds.	Activates human neutrophil integrin function, migration, and ROS production in vitro. Accelerates closure of MRSA-infected diabetic wounds. No acceleration of closure was observed in uninfected diabetic wounds.	Preclinical	[128]
PL-5/Peceleganan	Ac-KWKSFLKTFKSAAKTVLHTALKAISS-NH_2_	AMP selected through rational peptide design	Broad antibacterial activity against wound-associated Gram-positive and Gram-negative bacteria, including antibiotic-resistant isolates. Direct antibiofilm activity of free PL-5 was not evaluated in the cited studies.	No direct immunomodulatory or intrinsic pro-reparative activity has been established. Wound-related outcomes have been evaluated clinically.	Clinical	[129,130,131,132].

**Table 4:** Published and registered clinical evaluation of topical AMP formulations in DFU/DFI and other chronic or infected wounds.

AMP Formulation	Clinical Setting	Trial Identifier and Phase	Study Design and Intervention	Clinical Outcome	Microbiological and Biomarker Findings	Safety and Systemic Exposure	Reference
LL-37 topical formulation	Hard-to-heal VLUs	EudraCT: 2012-002100-41, phase I/II	Randomized, double-blind, placebo-controlled first-in-human trial, *n* = 34. LL-37 at 0.5, 1.6, or 3.2 mg/mL applied twice weekly for 4 weeks vs. placebo	Healing rate was significantly increased with 0.5 mg/mL and numerically increased with 1.6 mg/mL; 3.2 mg/mL showed no benefit over placebo	Microbiological efficacy was not a principal endpoint	No major local or systemic safety concerns. Treatment was generally well tolerated	[145]
		HEAL LL-37; EudraCT: 2018-000536-10, phase IIb	Multicenter, randomized, double-blind, placebo-controlled trial, *n* = 149 randomized (148 treated). LL-37 at 0.5 or 1.6 mg/mL vs. placebo, administered twice weekly for up to 13 weeks	No significant improvement in the primary endpoint of confirmed complete wound closure or in other healing outcomes in the overall cohort. Post hoc analysis suggested benefit of 0.5 mg/mL in patients with ulcers ≥ 10 cm^2^	Microbiological efficacy was not a primary endpoint. An exploratory substudy assessed LL-37 levels in wound exudate	Both dose levels were well tolerated and showed a favorable safety profile	[146]
LL-37 cream	DFU, uninfected or mildly infected ulcers were eligible and most enrolled ulcers were uninfected	NCT04098562, phase II	Randomized, placebo-controlled, parallel-group, double-blind trial, *n* = 25 (13 LL-37, 12 placebo). LL-37 cream at 0.5 mg/g twice weekly for 4 weeks vs. placebo; both groups received standard wound debridement	Granulation index increased significantly with LL-37 at weeks 1–4. Reduction in wound area was numerically greater but not significantly different from placebo	No significant between-group differences in aerobic bacterial burden or wound-fluid IL-1α and TNF-α levels	No adverse effects were directly attributed to LL-37	[109]
Pexiganan acetate cream (MSI-78)	Mildly infected DFU	NCT00563394 (study 303) and NCT00563433 (study 304), phase III	Two consecutive randomized, double-blind, multicenter active-controlled trials, combined *n* = 835. Topical pexiganan cream vs. oral ofloxacin, with matched topical and oral placebos	Study 303 failed to demonstrate equivalence; study 304 and pooled analysis showed similar clinical improvement (85–90%) and wound-healing outcomes between pexiganan and ofloxacin	Pooled microbiological eradication rates were similar between treatments (42–47%). Resistance to ofloxacin emerged in some patients, whereas no significant resistance to pexiganan was detected	Worsening cellulitis (2–4%) and amputation (2–3%) did not differ significantly between treatment groups	[147]
Pexiganan cream (Locilex)	Mildly infected DFU	NCT01590758 (OneStep-1) and NCT01594762 (OneStep-2), phase III	Two randomized, double-blind, multicenter, superiority, placebo-controlled trials. Pexiganan 0.8% twice daily for 14 days plus standard wound care vs. placebo plus standard wound care; *n* = 189 and *n* = 200, respectively	Neither trial met the primary endpoint of superiority for clinical resolution of infection. In OneStep-1, clinical response was 50.6% with pexiganan vs. 60.6% with placebo	Neither trial demonstrated superior bacterial eradication. In OneStep-1, microbiological success was 27.1% vs. 36.8%	TEAEs were monitored. Registry data do not establish a favorable efficacy–safety advantage	[148,149]
PL-5 (Peceleganan) spray	Secondary skin wound infections, including Wagner grade 2 DFU	ChiCTR2000033334, phase IIb	Multicenter, randomized, open-label, active-controlled trial, *n* = 220. PL-5 at 1‰, 2‰, or 4‰ vs. 1% silver sulfadiazine cream	SIRS-based clinical efficacy rates were higher across PL-5 groups than with SSD at days 5 and 8	Bacterial clearance rates did not differ significantly between PL-5 groups and SSD at either day 5 or day 8	No severe treatment-related adverse events. PL-5 was not detectable in blood during pharmacokinetic assessment	[131]
	Heterogeneous secondary skin wound infections; DFU represented a small subgroup	ChiCTR2100047202, phase III	Multicenter, randomized, open-label, active-controlled, parallel-group trial, *n* = 570. PL-5 at 2‰ vs. 1% silver sulfadiazine cream	SIRS-based clinical efficacy was higher with PL-5 at day 8 (90.4% vs. 78.7%) and day 5 (59.2% vs. 49.2%)	Bacterial clearance was lower with PL-5 at day 8 (24.0% vs. 46.0%) and day 5 (16.5% vs. 30.7%)	Treatment-related adverse-event profiles were similar between groups. No severe treatment-related adverse events were reported	[130]
	Mild-to-moderate infected DFU	ChiCTR2300068632, phase not specified	Multicenter, randomized, double-blind, placebo-controlled parallel group trial, *n* = 48 (33 PL-5, 15 placebo). PL-5 at 2‰ once daily plus standardized debridement vs. placebo plus debridement	Clinical infection response was higher with PL-5 (90.0% vs. 73.3%) at EOT1 and EOT7. Wound-area improvement did not differ significantly between groups at EOT1. A higher wound-healing rate was reported at EOT7.	Overall microbiological eradication did not differ significantly at EOT1 (57.89% vs. 33.33%) or EOT7 (64.71% vs. 40.00%). Higher clearance was reported in the drug-resistant subgroup	Adverse-event rates were 24.24% vs. 33.33%. No serious drug-related adverse events	[132]
	Mild DFI/infected DFU, IWGDF grade 2	NCT06189638, phase II	Randomized, double-blind, placebo-controlled multicenter trial, planned *n* = 90, 1:1:1 allocation. PL-5 1‰ or 2‰ vs. placebo twice daily for 14 days	Results not yet reported; primary endpoint is clinical response at end of therapy	Secondary endpoints include microbiological response, comprehensive clinical–microbiological response, wound infection score, and recurrence or follow-up response	Safety and tolerability are prespecified outcomes	[150]
	Wound infections; not DFU-specific	ChiCTR2300071255, phase IIIb	Multicenter, randomized, double-blind, placebo-controlled wound-infection trial	Peer-reviewed outcome data were not identified	N/A	Safety and systemic wound-to-blood exposure are included in the registered development program	[151]

Abbreviations: SIRS, Skin Infection Rating Scale; EOT, end of treatment; TEAE, treatment-emergent adverse event. Pexiganan (MSI-78/Locilex) and Peceleganan (PL-5) are distinct AMP candidates. Registry-only studies are included to indicate the current stage of clinical development; absence of reported outcomes should not be interpreted as evidence of efficacy or failure.

## Data Availability

No new data were created or analyzed in this study. Data sharing is not applicable to this article.

## Abbreviations

The following abbreviations are used in this manuscript:

AGEs	advanced glycation end products
AMP	antimicrobial peptide
ATP	adenosine triphosphate
BLT1	leukotriene B4 receptor 1
DFI	diabetic foot infection
DFU	diabetic foot ulcer
DM	diabetes mellitus
ECM	extracellular matrix
ESBL	extended-spectrum β-lactamase
hBD	human β-defensin
hCVAM	human cryopreserved viable amniotic membrane
HNP	human neutrophil peptide
Hst	histatin
HUVEC	human umbilical vein endothelial cell
LTB4	leukotriene B4
MDR	multidrug resistance
MMP	matrix metalloproteinase
MRSA	methicillin-resistant *S. aureus*
MSSA	methicillin-sensitive *S. aureus*
NETs	neutrophil extracellular traps
PDGF-BB	platelet-derived growth factor-BB
RAGE	receptor for advanced glycation end products
ROS	reactive oxygen species
STZ	streptozotocin
T1DM	type 1 diabetes mellitus
T2DM	type 2 diabetes mellitus
TIMP	tissue inhibitor of metalloproteinase
VEGF	vascular endothelial growth factor
VLU	venous leg ulcer
α-SMA	α-smooth muscle actin
